# Unifying Phytochemistry, Analytics, and Target Prediction to Advance *Dendropanax morbifera* Bioactive Discovery

**DOI:** 10.3390/life16010100

**Published:** 2026-01-11

**Authors:** SuHyun Kim, Damhee Lee, Kyujeong Won, Jinseop Lee, Wooseop Lee, Woohyeon Roh, Youngjun Kim

**Affiliations:** Department of Medicinal Bioscience and Nanotechnology Research Center, Konkuk University, Chungju 27478, Chungbuk, Republic of Korea; bb05183@naver.com (S.K.); davil234@naver.com (D.L.); wkj0917@kku.ac.kr (K.W.); mm7476361m@naver.com (J.L.); dldntjq7000@kku.ac.kr (W.L.); shdngus01@naver.com (W.R.)

**Keywords:** bioactive compounds, *Dendropanax morbifera*, HPLC profiling, phytochemistry, target prediction

## Abstract

*Dendropanax morbifera* (DM; “Hwangchil”) is an evergreen tree native to southern Korea and Jeju Island, traditionally used for detoxification, anti-inflammatory, immunomodulatory, and neuroprotective purposes. Recent studies indicate that DM extracts and their constituents exhibit a broad range of biological activities, including antioxidant, anti-inflammatory, antimicrobial, anticancer, antidiabetic, hepatoprotective, and neuroprotective effects. Phytochemical investigations have revealed a chemically diverse profile comprising phenolic acids, flavonoids, diterpenoids, triterpenoids—most notably dendropanoxide—and polyacetylenes, with marked variation in compound distribution across plant parts. Despite this progress, translational application remains constrained by the lack of standardized extraction protocols, substantial variability in high-performance liquid chromatography (HPLC) methodologies, and limited mechanistic validation of reported bioactivities. This review proposes an integrated framework that links extraction strategies tailored to compound class and plant part with standardized C18 reverse-phase HPLC conditions to enhance analytical reproducibility. In parallel, in silico target prediction using SwissTargetPrediction is applied as a hypothesis-generating approach to prioritize potential molecular targets for subsequent experimental validation. By emphasizing methodological harmonization, critical evaluation of evidence levels, and systems-level consideration of multi-compound interactions, this review aims to clarify structure–activity relationships, support pharmacokinetic and safety assessment, and facilitate the rational development of DM-derived materials for medical, nutritional, and cosmetic applications.

## 1. Introduction

*Dendropanax morbifera* (DM) is an evergreen tree of the Araliaceae family indigenous to East Asia, with a high prevalence in the southern coastal regions of the Korean Peninsula and Jeju Island [1,2]. The plant has been extensively utilized in traditional Korean medicine for its detoxifying, anti-inflammatory, immunomodulatory, and neuroprotective properties, and various plant parts—including leaves, stems, roots, and sap—have been employed as medicinal materials [1,2,3,4,5]. Referred to as “Hwangchil,” DM has additional cultural importance due to its historical application as a natural lacquer. This rich ethnopharmacological heritage has underpinned growing scientific efforts to elucidate its phytochemical composition and pharmacological potential [1,6]

Over the last several decades, DM has been recognized for its broad pharmacological potential. Numerous studies have reported antioxidant, anti-inflammatory, antimicrobial, anticancer, neuroprotective, antidiabetic, and hepatoprotective activities in DM extracts and constituents [7,8,9,10,11,12,13,14,15,16,17,18,19,20]. These findings highlight DM as a promising candidate for applications in pharmaceuticals, functional foods, and cosmetics [7,17,21,22,23,24,25]. In parallel with the global rise in plant-based bioactive research, DM has emerged as an important model reflecting the integration of traditional knowledge and modern evidence-based evaluation [5,7].

Phytochemical analyses indicate that DM contains a wide spectrum of metabolites, including flavonoids, phenolic acids, triterpenoids, coumarins, lignans, steroids, and polyacetylenes [22,26,27]. These compounds contribute to various biological effects, especially antioxidant and anti-inflammatory activities, and modulate multiple molecular pathways linked to disease prevention and therapeutic efficacy [7,22,26,28]. Representative constituents include rutin and quercetin, which are well-known flavonoid antioxidants, and dendropanoxide, a characteristic diterpenoid implicated in AMP-activated protein kinase (AMPK) regulation [29,30,31,32,33]. Owing to this chemical diversity, DM provides a versatile platform for discovering natural molecules with relevance to medicine, health-promoting foods, and cosmetic formulations.

Despite these advantages, significant challenges remain in advancing DM research. A major limitation is the absence of standardized extraction, purification, and analytical protocols [34,35]. Variations in extraction conditions—including solvents, temperatures, and processing times—lead to inconsistent yields and phytochemical profiles, hindering reproducibility across studies. Analytical variability is further evident in high-performance liquid chromatography (HPLC)–based profiling, where differences in mobile-phase composition, column selection, gradient design, and detection settings restrict cross-study comparability [34,36]. In addition, inconsistent use of complementary structural tools, such as mass spectrometry and nuclear magnetic resonance, has led to discrepancies in compound identification and characterization, complicating the establishment of consensus chemical profiles [27].

Another notable gap concerns mechanistic understanding. Although many studies have explored the biological activities of DM extracts or isolated compounds, most have remained at the in vitro screening stage [19,37,38]. Mechanistic validation, including the identification of molecular targets and confirmation of direct ligand–target interactions, remains limited. Even for promising compounds such as dendropanoxide, robust biophysical evidence for target engagement and detailed pharmacokinetic characterization is lacking. Furthermore, a systematic ligand–target mapping approach for major DM constituents has not yet been fully developed, limiting the translation of phytochemical findings into rational, hypothesis-driven research and development. It is crucial to differentiate between hypothesized and validated mechanisms. For instance, while in silico docking studies propose potential targets, only further experimental validation, such as surface plasmon resonance (SPR), can confirm these findings. Clear labeling of ‘putative’ versus ‘validated’ interactions in the literature would help prevent the conflation of preliminary data with confirmed scientific evidence. At the same time, it should be acknowledged that pharmacokinetic properties, bioavailability, and achievable exposure levels of DM-derived phytochemicals remain insufficiently characterized, representing important challenges for translating preclinical findings into practical applications.

To address these issues, this review proposes an integrated strategy that combines phytochemical standardization, analytical harmonization, and molecular target mapping. Specifically, we outline standardized HPLC conditions designed to improve reproducibility and comparability; present a ligand–protein interaction framework linking key DM-derived compounds to major molecular targets such as sirtuin 1, sirtuin 6, AMPK, cannabinoid receptors, the pregnane X receptor, and cytochrome P450 enzymes; and introduce a research roadmap involving plant-part-specific extraction procedures, compound-class-guided chromatographic optimization, biophysical validation, pharmacokinetic studies, and structure–activity relationship (SAR) investigations [27,36]. The molecular targets highlighted in this review were defined through converging evidence linking the reported bioactivities of DM to central regulatory hubs of metabolism, inflammation, and xenobiotic homeostasis. These targets—frequently modulated by polyphenols and terpenoids abundant in DM—provide the most mechanistically informative framework for translating DM-specific chemistry into biologically and clinically relevant insights.

Moreover, to foster broader adoption of this roadmap, we propose specific metrics to gauge progress and success. Firstly, achieving an inter-laboratory relative standard deviation (RSD) of less than 5% in the quantification of rutin across different labs would serve as a benchmark for analytical consistency. Secondly, establishing a target to elucidate at least three new ligand–target interactions for DM-derived compounds using both experimental and computational methods within the next three years will highlight the practical applicability and innovation enabled by this integrated approach.

In summary, this review brings together information on DM’s chemistry, analysis, and mechanisms of action to help guide future research. Our goal is to support the systematic development of DM-based compounds for use in medicine, nutrition, and cosmetics.

## 2. Phytochemical Profile and Bioactivities

**Figure 1:**
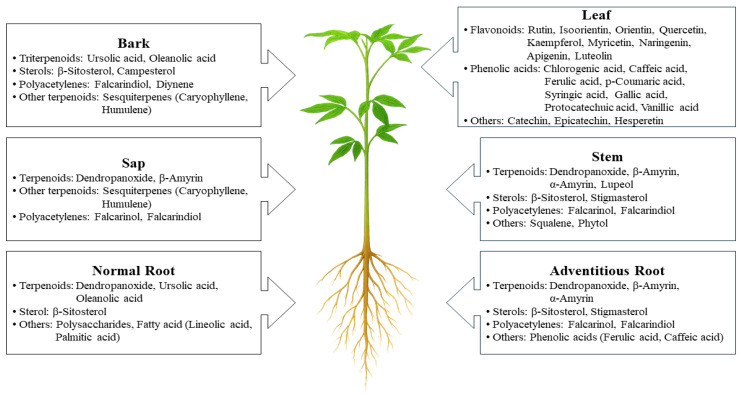
Phytochemical distribution across different parts of *Dendropanax morbifera*. Leaves predominantly contain flavonoids and phenolic acids, whereas stems exhibit a mixed profile of secondary metabolites. Roots are enriched in polyacetylenes and triterpenoids, while the sap is characterized by a high abundance of triterpenoids, including dendropanoxide. Only representative, well-characterized compounds are presented.

*Dendropanax morbifera* (DM) displays pronounced phytochemical diversity that reflects both ecological adaptation and its promise as a source of bioactive natural products [1,27]. Phytochemical studies have identified diverse classes of secondary metabolites, including phenolic acids, flavonoids, terpenoids (triterpenoids and diterpenoids), polyacetylenes, lignans, and quinic acid derivatives, which are differentially accumulated in the leaves, stems, roots, and sap [35,39,40,41,42,43]. This organ-specific chemical distribution (Figure 1) likely represents an adaptive chemical defense strategy evolved in response to biotic and abiotic stresses, such as microbial infection, herbivore pressure, and oxidative stress [2,39,44].

Phenolic acids and flavonoids, particularly chlorogenic acid and rutin, are among the most abundant constituents [13,33,45]. These metabolites are well known for their potent antioxidant and anti-inflammatory properties, indicating that DM has evolved biochemical strategies to regulate redox homeostasis and protect tissues against oxidative injury [39,44,46,47]. Flavonoids such as quercetin and myricetin may additionally contribute to photoprotection, an adaptive advantage for a species native to sun-exposed regions of southern Korea and Jeju Island [47]. The prevalence of caffeic acid and its derivatives further implies a role in antimicrobial defense, consistent with traditional uses of DM as an antimicrobial remedy [3,4].

The presence of distinctive terpenoids, such as dendropanoxide, and triterpenoids, including α- and β-amyrin, underscores the metabolic specialization of DM [8,30,31]. These lipophilic molecules, frequently enriched in the cuticular layer and resinous sap, function not only as structural or protective agents but also as chemical barriers against environmental stressors. Their pharmacological relevance extends well beyond ecological roles: dendropanoxide has been reported to modulate AMPK signaling and exhibit antidiabetic, nephroprotective, and anti-inflammatory activities in preclinical models [8,18,31], while α- and β-amyrin demonstrate robust anti-inflammatory effects in murine cell line RAW 264.7 macrophages [15,46].

Polyacetylenes and rare glycosides identified in DM add yet another dimension to its ecological and pharmacological profile [41,48]. These metabolites, common in plants inhabiting harsh or competitive environments, may function as deterrents against herbivory or as mediators of plant–microbe interactions. Their biological relevance is supported by evidence such as the anticomplement activity of falcarinol and falcarindiol [2], and the neuroprotective properties of syringin and isoquercitrin in neuronal cell models [49,50,51].

As summarized in Table 1, these phytochemicals exhibit not only substantial structural diversity but also a wide array of experimentally validated bioactivities. These include antioxidant (e.g., quercetin in Human Keratinocyte Cell Line (HaCaT) keratinocytes; syringin in 2,2-diphenyl-1-picrylhydrazyl (DPPH) radical assays), anti-inflammatory (e.g., 3,5-dicaffeoylquinic acid and (+)-catechin in RAW 264.7 macrophages), antidiabetic (e.g., dendropanoxide in streptozotocin-induced diabetic rats), cognitive-enhancing (e.g., orientin and isoorientin), neuroprotective (e.g., isoquercitrin and quercetin in Mouse Hippocampal Neuronal (HT22) cells), and hepatoprotective effects (e.g., hydroxyl radical scavenging activity (HOD) in Human Hepatocellular Carcinoma (HepG2) cells). A comparative analysis reveals that while quercetin alone exhibits notable antioxidant activity, the combination of quercetin with caffeic acid significantly enhances these effects. For instance, an oxidative assay showed that the IC_50_ of quercetin decreased when combined with caffeic acid, indicating a synergistic interaction between the two compounds [35]. This case illustrates how phytochemical synergy can amplify the biological functions of DM constituents. Together, the table provides an integrated overview linking specific chemical constituents to their documented biological functions.

It should be noted that the bioactivities summarized above are supported by evidence derived from different experimental levels, which vary in their translational implications. A substantial proportion of the reported antioxidant and enzyme-related effects are based on chemical or biochemical assays, such as radical scavenging or in vitro inhibition tests [7,16,35,36,54], which provide useful initial indications of activity but do not account for cellular uptake or biological complexity. These findings are complemented by cell-based studies [16,19,40,41,46,52,53,54,56,57] that offer mechanistic insights under controlled conditions, while a more limited number of investigations extend to animal models [8,18,49,55], where systemic exposure and physiological relevance can be assessed. Clear differentiation among these levels of evidence is essential for appropriately interpreting the strength of current findings and for framing the challenges associated with translating DM-derived phytochemicals into practical applications.

**Figure 2:**
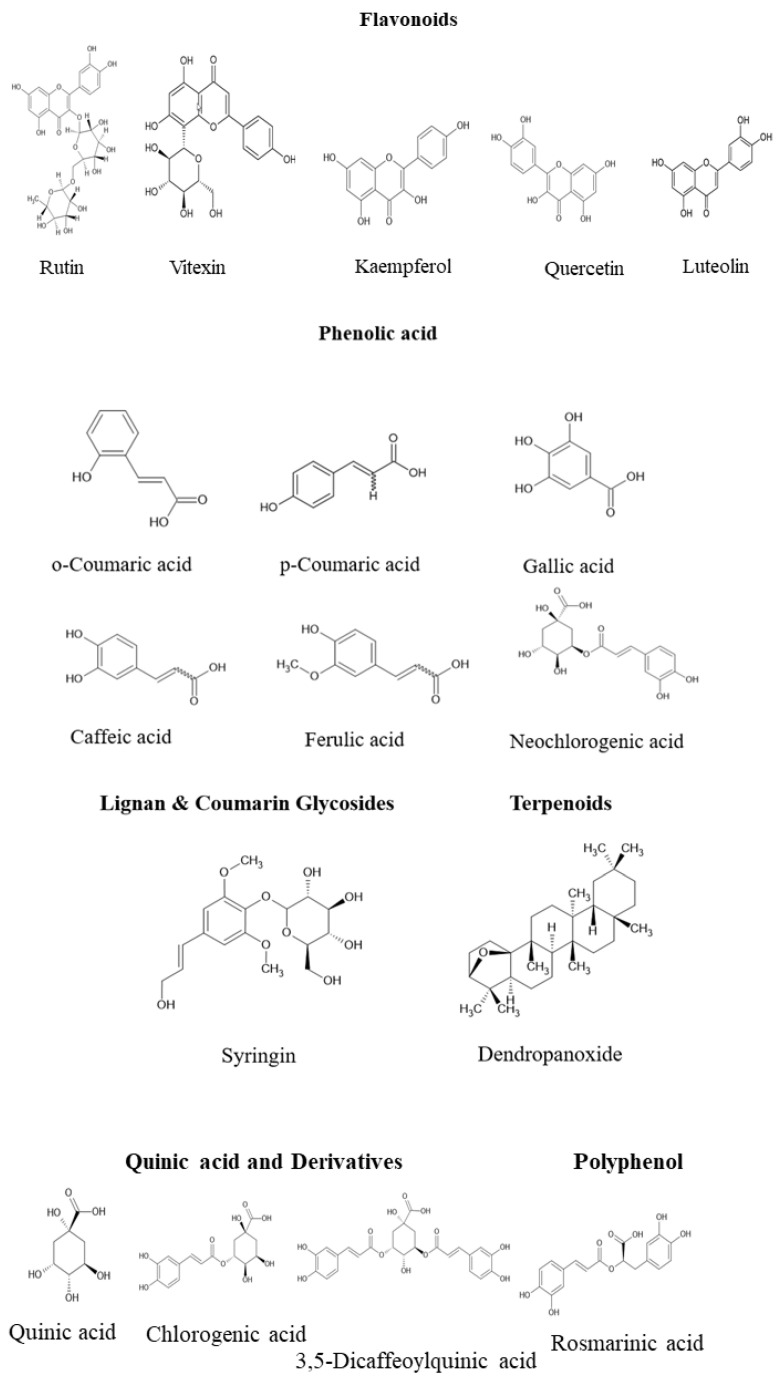
Representative chemical structures of major bioactive compounds isolated from *Dendropanax morbifera*. The figure highlights key flavonoids, phenolic acids, diterpenoids, polyacetylenes, and other secondary metabolites that collectively contribute to the plant’s diverse pharmacological activities.

As illustrated in Figure 1, the metabolite profiles vary significantly among plant parts: leaves and stems are enriched in flavonoids and phenolic acids, supporting antioxidant defense, whereas roots contain higher levels of polyacetylenes and selected triterpenoids, potentially related to subterranean microbial interactions. The sap is distinguished by its diterpenoid abundance—including dendropanoxide—which serves as a chemical hallmark of DM [2,31,39,41,44]. Representative chemical structures are depicted in Figure 2, highlighting the structural heterogeneity underlying the diverse bioactivities of DM metabolites [1,27].

Overall, the diverse chemical composition of DM substantiates its traditional medicinal applications and illustrates the plant’s adaptive evolution. Examination of the data in Table 1 clarifies how individual compounds and their combined effects contribute to both plant survival and human health benefits. This perspective positions DM not merely as a source of isolated compounds, but as a complex system with significant potential for novel therapeutic development [1,31,44,49,59].

## 3. Extraction Strategies and Methodological Variability

The extraction of bioactive compounds from *Dendropanax morbifera* (DM) has been extensively investigated (Tables S1 and S2); however, substantial variability persists across studies in solvent systems, plant parts, and extraction parameters [2,27,34,56,60]. Polar solvents—including water (H_2_O), ethanol (EtOH), and methanol (MeOH)—are most commonly employed due to their high efficiency in extracting phenolic acids, flavonoids, and other hydrophilic metabolites [10,14,24,35,39,44,61]. For instance, 70–80% EtOH is frequently used for leaf extraction and consistently yields high levels of rutin and chlorogenic acid [35,39], whereas methanol-based protocols are often associated with enhanced recovery of diterpenoids such as dendropanoxide [30,31,34,62,63]. This alignment between solvent polarity and metabolite solubility underscores the central role of solvent selection in determining phytochemical composition [34,35,39,56].

Plant-part-specific extraction further contributes to optimizing chemical diversity [2,26,27,58]. Leaf tissues are particularly rich in phenolic acids and flavonoids, which account for strong antioxidant and anti-inflammatory activity [10,35,39], while the bark and roots contain higher levels of triterpenoids and polyacetylenes, including dendropanoxide—a distinguishing marker compound of DM [26,27,30,31,58]. In addition, aqueous extraction efficiently isolates highly polar metabolites such as syringin, which is especially relevant for food-grade or pharmaceutical applications where the Generally Recognized as Safe (GRAS) status of water provides a regulatory advantage [1,21,50].

Despite progress in extraction methodologies, critical limitations persist. First, the absence of standardized protocols has resulted in considerable inconsistency across studies, with variations in solvent concentration, extraction duration, temperature, and sample preparation complicating meaningful cross-comparison. Second, the predominant reliance on polar solvents has led to the underrepresentation of lipophilic metabolites—particularly triterpenoids and polyacetylenes—due to insufficient optimization of non-polar or sequential partitioning techniques. Third, the limited integration of extraction research with biological activity assays restricts the ability to systematically associate extraction strategies with pharmacological outcomes.

To address these issues, this review proposes standardized extraction conditions tailored to specific compound classes and plant parts. By aligning solvent polarity, extraction techniques, and analytical workflows, these recommendations aim to enhance reproducibility, support cross-study comparability, and enable targeted exploration of DM’s chemical and biological potential. As summarized in Table 2, ethanol- and methanol-based extractions remain indispensable for enriching phenolic- and flavonoid-dominant fractions, whereas partitioning-based approaches are more suitable for isolating lipophilic constituents such as polyacetylenes and triterpenoids. Water-based extractions offer a safe, scalable approach for highly polar compounds, enabling applications in functional foods and pharmaceutical formulations. To harmonize these protocols, it is crucial to conduct inter-laboratory studies and develop shared standard operating procedures (SOPs) that can be widely adopted. Coordinated efforts among research facilities can foster consistency, with labs collaboratively verifying protocol effectiveness and sharing results through established networks. Such measures will significantly advance standardization efforts in the field, facilitating broader acceptance and application of these methodologies.

In addition to methodological variability, the thermal and oxidative instability of certain DM constituents should be considered, as phenolic acids, flavonoids, and polyacetylenes may degrade under elevated temperatures or prolonged extraction conditions, thereby altering phytochemical profiles and apparent bioactivities. Moreover, increasing regulatory and sustainability-driven demands are promoting the use of greener solvents, such as aqueous ethanol, particularly for food and pharmaceutical applications, underscoring the need to evaluate how these approaches influence extraction efficiency, compound stability, and biological outcomes in DM.

Importantly, early extraction choices have direct consequences for the reproducibility of downstream biological and mechanistic findings. Variations in solvent systems, temperature, and processing conditions can selectively enrich or deplete specific metabolite classes, thereby shaping the apparent bioactivity profiles observed in cell-based or in vivo assays. As a result, inconsistencies in extraction protocols may propagate into divergent biological outcomes and mechanistic interpretations, even when similar plant materials are used. Explicitly linking extraction strategies to subsequent bioassay performance is therefore essential for achieving reliable, mechanism-oriented evaluation of DM-derived compounds.

The establishment of standardized extraction methods will enhance consistency in phytochemical analyses and enable reliable identification and confirmation of active compounds in DM by linking chemical profiles to biological effects.

## 4. Standardized HPLC Strategies for Phytochemical Profiling

High-performance liquid chromatography (HPLC) is the most widely employed analytical technique for the phytochemical characterization of *Dendropanax morbifera* (DM). Literature surveys (Table S3) indicate that more than 80% of published studies utilize reverse-phase C18 columns [64], typically with lengths of 150–250 mm, internal diameters of 2.0–4.6 mm, and particle sizes of 3–5 μm. This configuration has demonstrated reliable separation performance for major metabolite classes—including phenolic acids, flavonoids, diterpenoids, triterpenoids, and polyacetylenes [34,46,60]. Although alternative stationary phases such as C8 or phenyl-hexyl columns have been used in select cases to enhance the retention of highly lipophilic constituents, these instances remain uncommon, and C18 phases are firmly established as the analytical standard [2,17,38,60].

Mobile phase compositions reported in the literature exhibit substantial diversity yet follow consistent principles. Most methods employ binary systems in which the aqueous phase consists of water acidified with 0.05–0.1% formic acid (FA)or acetic acid (AcOH), paired with acetonitrile (ACN) or methanol (MeOH) as the organic modifier. Acidification improves peak shape, minimizes tailing, and enhances ionization efficiency for MS-coupled detection [34,65]. ACN-based gradients are generally preferred for phenolic acids and flavonoids due to improved resolution and lower baseline noise, whereas MeOH is sometimes favored for triterpenoids and polyacetylenes because its higher viscosity increases retention of nonpolar compounds [11,26,35,58].

Gradient programs vary widely but typically span 30–60 min with organic solvent compositions increasing from 10% to 90% [66]. For phenolic-rich fractions, gradients often begin at 5–10% organic solvent and progress to 40–60% within 20–30 min, enabling efficient separation of chlorogenic acid, rutin, and other hydrophilic metabolites. In contrast, the analysis of triterpenoids and polyacetylenes requires extended gradients reaching 80–90% organic solvent to elute highly lipophilic constituents such as dendropanoxide and (3S)-falcarinol. Some studies (Table S3) have incorporated step-gradient transitions to improve the resolution of flavonoid glycosides versus aglycones, thereby reducing co-elution and improving quantification precision.

Detection strategies have evolved substantially with advances in analytical technologies. UV and diode-array detection (DAD) remain foundational for initial profiling, with optimized wavelength ranges applied according to chemical class: 320–330 nm for phenolic acids, 254–280 nm for flavonoids, and 210–254 nm for diterpenoids and triterpenoids [34]. However, the inherent limitations of UV detection become evident for compounds with weak chromophores, including dendropanoxide and several polyacetylenes. As a result, tandem liquid chromatography–mass spectrometry (LC-MS/MS)—particularly quadrupole time-of-flight mass spectrometry (QTOF-MS) and triple-quadrupole instruments—has been widely adopted for structural confirmation and enhanced sensitivity. Additionally, charged aerosol detectors (CAD) and evaporative light-scattering detectors (ELSD) have been successfully integrated into workflows for the analysis of triterpenoids and saponin-like components [34].

Despite significant methodological progress, variability in chromatographic conditions remains a major challenge. Differences in column dimensions, mobile-phase systems, gradient profiles, and detector configurations lead to inconsistent retention times and unreliable quantification across laboratories [17,67]. Furthermore, many studies lack essential validation parameters—such as calibration curves, limits of detection (LOD), and limits of quantification (LOQ)—thereby limiting the robustness of reported phytochemical data.

To address these challenges, standardized HPLC conditions for DM analysis are recommended, as summarized in Table 3. These include compound class–specific gradient programs, optimized detection wavelengths, and the use of mass spectrometry or alternative detectors for compounds with weak UV absorbance. Adoption of these standardized conditions will improve inter-laboratory reproducibility and facilitate correlation between chemical profiles and biological effects, thereby supporting the reliable use of DM as a source of bioactive compounds for medical, nutritional, and related applications.

In this context, chromatographic fingerprinting is generally sufficient for quality control, batch-to-batch comparison, and comparative evaluation of extraction conditions, where relative peak patterns and overall chemical consistency are the primary objectives. In contrast, absolute quantification of individual DM constituents is required for mechanistic studies, dose–response evaluations, and regulatory applications, in which precise concentration data are essential to ensure biological reproducibility and meaningful interpretation. From an industrial and translational perspective, combining chromatographic fingerprinting with marker-based quantification enables robust batch-to-batch quality control, ensures chemical consistency, and supports the scalable development of DM-derived products.

We emphasize that the objective of extraction standardization for DM is not to achieve an absolutely uniform chemical composition across all batches—which is unrealistic given inherent variability arising from climatic, seasonal, and geographical factors—but rather to ensure controlled, purpose-oriented reproducibility. In this context, we propose a two-tier standardization framework.

First, extraction conditions should be standardized at the process level, including the solvent system (e.g., aqueous ethanol within a defined polarity range), solvent-to-solid ratio, extraction temperature, extraction time, and number of extraction cycles. These parameters should be selected and optimized according to the intended application, such as phenolic-enriched extracts for functional food development or diterpenoid-focused fractions for mechanistic research.

Second, batch consistency should be ensured at the chemical outcome level by defining acceptance criteria based on chromatographic fingerprints in combination with a limited set of quantitatively monitored marker compounds (e.g., representative phenolic acids, flavonoids, and dendropanoxide). This approach is consistent with established quality-by-design principles in botanical drug and functional food development, where reproducibility is defined by controlled process parameters and acceptable compositional ranges rather than absolute chemical identity.

## 5. Ligand–Target Mapping of Bioactive Compounds

To predict plausible macromolecular targets of bioactive compounds, SwissTargetPrediction (STP) was employed. STP [68,69] estimates target likelihoods based on combined two-dimensional (2D) and three-dimensional (3D) structural similarity between query molecules and curated libraries of bioactive ligands. Summaries of reported biological activities and mechanisms of action for representative compounds isolated from DM are provided in Table S4 (rutin and chlorogenic acid) and Table S5 (other representative compounds). Among DM-derived phytochemicals, six representative compounds with well-characterized protein target interactions were selected, including four compounds (quercetin, kaempferol, luteolin, and ferulic acid) for comparative analysis and two reference compounds (resveratrol and cannabidiol (CBD)) for benchmarking. As summarized in Table 4, these phytochemicals were systematically evaluated for predicted protein interactions and associated biological functions based on integrated considerations of molecular pharmacology, structural features, and reported bioactivities.

The data presented in Table 4 do not exclusively reflect activities derived from compounds directly isolated and experimentally tested from DM extracts. Instead, Table 4 integrates two complementary evidence streams: (i) DM-derived phytochemicals experimentally identified in *Dendropanax morbifera* with available target information [16,70,71,72,73,74,75,76], and (ii) well-characterized reference phytochemicals whose molecular targets and biological activities have been extensively validated in the literature [66,70,77] and are included for comparative and benchmarking purposes. Although these compounds possess broader target spectra than those listed, the table focuses on a curated subset of targets most relevant to the disease contexts and mechanistic pathways discussed in this study.

Overall, the results in Table 4 indicate that these phytochemicals act through multi-target mechanisms rather than single molecular pathways. Each compound modulates multiple enzyme and receptor networks, a feature relevant to complex diseases such as neurodegenerative disorders, chronic inflammation, and metabolic diseases [78,79]. To complement experimentally supported interactions, STP analysis was additionally conducted for the *D. morbifera*–derived ligands examined in this study (Supplementary Materials). Predicted targets were grouped into major functional classes (Table 5), and the complete list of predicted targets with corresponding probability scores is provided in the Supplementary File (STP_results.xls).

STP analysis identified kinases, oxidoreductases, carbonic anhydrases, G protein–coupled receptors, proteases, and nuclear receptors as dominant predicted target categories. Kinases were the most prevalent class (n ≈ 45; average probability 0.45–0.65), followed by oxidoreductases (n ≈ 20) and lyases, particularly carbonic anhydrase isoforms (CA1–CA14; n ≈ 15; probability range 0.40–0.80). The enrichment of carbonic anhydrases suggests potential involvement in pH regulation and tumor microenvironment adaptation. High-probability targets included protein kinase B alpha (AKT1), glycogen synthase kinase 3 beta (GSK3B), spleen tyrosine kinase (SYK), matrix metalloproteinase 9 (MMP9), beta-site APP-cleaving enzyme 1 (BACE1), cytochrome P450 1B1 (CYP1B1), and multiple carbonic anhydrase isoforms, consistent with previously proposed mechanisms underlying the biological activities of *D. morbifera* constituents.

To evaluate the reliability of the in silico predictions, a concordance analysis was performed by comparing STP outputs with literature-validated protein targets of well-characterized phytochemicals (Table 6). Across quercetin, kaempferol, resveratrol, luteolin, ferulic acid, and CBD, STP recovered approximately 50–70% of experimentally established targets, indicating substantial agreement with known binding profiles [34]. This concordance supports the utility of STP as a hypothesis-generating tool for prioritizing plausible molecular targets of *D. morbifera* constituents.

**Table 4:** Literature-validated molecular targets and binding evidence for representative phytochemicals isolated from *Dendropanax morbifera*.

Compound	Protein Targets *	Binding Evidence	Reference
Cannabidiol (CBD)	CB1; CB2	Radioligand binding assay	[66,77]
Resveratrol	COX2; ESR1	Cell signaling assay; In silico docking	[70]
Quercetin	SIRT1; AChE; AKT1	Enzymatic assay; In silico docking	[71,72,73]
Kaempferol	AKT1; MMP9; GSK3B	Direct binding assays; In silico docking	[70,74]
Luteolin	PTPRZ1; STAT3	Molecular dynamics; In silico docking	[16,75]
Ferulic acid	PTPRZ1; NOS2	Molecular dynamics; In silico docking	[75,76]

* Protein targets and activities summarized in this table are derived from the broader literature on each compound and are not restricted to interactions experimentally validated specifically in *Dendropanax morbifera* extracts. Only representative, well-documented targets relevant to the scope of this review are shown. In silico results should be interpreted as hypothesis-generating and require experimental validation.

CBD was used as a primary validation standard because its canonical targets, cannabinoid receptor 1 (CB1) and cannabinoid receptor 2 (CB2), are well established through radioligand binding assays [66,77,80,81,82,83,84]. STP successfully recovered these interactions with a high probability score (0.893). Similarly, resveratrol yielded maximum probability scores (1.000) for cyclooxygenase-2 (COX2) and estrogen receptor α (ESR1), consistent with its established roles in metabolic regulation and anti-inflammatory signaling [70,85].

Among major DM constituents, quercetin showed an exact match for AKT1 (probability = 1.000) and additional support for acetylcholinesterase (AChE; 0.680). Kaempferol was predicted to interact with MMP9 and GSK3B (0.658), as well as AKT1 (0.403), supporting its reported anti-inflammatory and signaling-modulatory activities [57,73,74,86,87,88,89,90]. Predictions with probability scores ≥0.6 were considered high-confidence interactions.

In contrast, predicted targets for luteolin and ferulic acid, including protein tyrosine phosphatase receptor type Z1 (PTPRZ1), signal transducer and activator of transcription 3 (STAT3), and inducible nitric oxide synthase 2 (NOS2), showed low STP probability scores (0.000–0.031). These results reflect the reliance of STP on known ligand–target similarity and suggest that these compounds may interact through novel or non-canonical mechanisms not currently represented in available databases. Accordingly, these interactions were classified as putative and require further experimental validation.

Taken together, the concordance analysis demonstrates that STP effectively recovers a substantial proportion of known targets for standard phytochemicals, and, as exemplified by dendropanoxide, can additionally generate de novo predictions with moderate confidence, identifying cytochrome P450 19A1 (CYP19A1), sonic hedgehog protein (SHH), and cytochrome P450 51A1 (CYP51A1) as plausible molecular targets with identical STP probability scores of 0.119, thereby providing a rational basis for subsequent mechanistic and experimental validation.

**Table 5:** Major predicted target classes and representative proteins identified by SwissTargetPrediction for the compounds isolated from *Dendropanax morbifera*.

Target Class	Representative Predicted Targets	Count (n)	Average Probability	Biological Relevance
**Kinase**	AKT1, FLT3, SRC, SYK, GSK3B, CDK1/2/5/6, EGFR, MET, NEK2, PLK1	~45	~0.45–0.65	Neuroinflammation; tumor signaling; PI3K–AKT and MAPK pathways
**Oxidoreductases**	MAOA, XDH, CYP1B1, ALOX5, ALOX12, ALOX15	~20	~0.55–0.70	Oxidative stress modulation
**Lyases** **(Carbonic anhydrases)**	CA1, CA2, CA3, CA4, CA6, CA7, CA9, CA12, CA13, CA14	~15	~0.40–0.80	pH regulation; tumor microenvironment adaptation
**GPCRs**	ADORA1, ADORA2A, DRD4, GPR35, AVPR2	~10	~0.50–0.80	Neurotransmission; neuroprotective signaling
**Proteases**	MMP2, MMP3, MMP9, MMP13, BACE1, Thrombin (F2)	~10	~0.40–0.65	Extracellular matrix (ECM) remodeling; glioma invasion
**Phosphatases**	PTPRS	1 (high-confidence)	0.61	Glioblastoma-associated phosphatase
**Nuclear Receptors**	ESR1, ESR2, ESRRA	3	~0.27–0.50	Hormone signaling; metabolic regulation
**Transporters/Efflux Proteins**	ABCB1, ABCC1, ABCG2	3	~0.40–0.50	Drug resistance; xenobiotic metabolism
**Cytochrome P450 family**	CYP19A1, CYP1B1	2	~0.40–0.65	Metabolic detoxification
**Miscellaneous Enzymes**	ALDH2, PARP1, MPO, GLO1	~10	~0.40–0.60	Oxidative and aldehyde detoxification

**Table 6:** Concordance between literature-validated molecular targets and SwissTargetPrediction (STP)-predicted targets for representative phytochemicals from *Dendropanax morbifera*. ND denotes not determined.

Compound	Literature-Validated Targets	STP-Predicted Targets	STP Probability
Cannabidiol (CBD)	CB1	CB1	0.893
	CB2	CB2	0.893
Resveratrol	COX2	COX2	1.000
	ESR1	ESR1	1.000
Quercetin	SIRT1		0.000
	AKT1	AKT1	1.000
	AChE	AChE	0.680
Kaempferol	AKT1	AKT1	0.403
	MMP9	MMP9	0.658
	GSK3B	GSK3B	0.658
Luteolin	PTPRZ1		0.000
	STAT3		0.000
Ferulic acid	PTPRZ1		0.000
	NOS2	NOS2	0.031

Future studies should integrate computational docking with experimental approaches, including surface plasmon resonance, isothermal titration calorimetry, and cell-based assays. Such combined strategies will facilitate validation of predicted interactions and support structure–activity relationship optimization and drug discovery efforts. It should be emphasized that in silico docking and STP-based analyses are exploratory and hypothesis-generating and do not alone demonstrate definitive ligand–protein binding.

## 6. Current Research and Future Directions

Research on *Dendropanax morbifera* (DM) has substantially expanded over the past decade, leading to the identification of a chemically diverse set of phytochemicals, including phenolic acids, flavonoids, triterpenoids, and the unique diterpenoid dendropanoxide [1,34,40,57,60,91]. Accumulating in vitro and in vivo studies suggest that DM extracts and selected constituents exert antioxidant [92], anti-inflammatory [40,46,56,93,94,95], metabolic [5], and neuroprotective effects [11,49,57]. Together, these findings position DM as a promising source of bioactive natural products for functional food and therapeutic development [20,45,74,76,93,96]. However, despite this progress, the overall evidence base remains fragmented, with substantial variability in experimental design and depth of mechanistic validation.

A central limitation across the DM literature is the lack of standardized extraction and analytical methodologies. Reported studies vary widely in solvent selection, extraction temperature and duration, fractionation strategies, and plant parts analyzed, resulting in marked discrepancies in compound yields and reported bioactivities. Polar solvents such as ethanol and methanol are commonly used for phenolic acids and flavonoids, whereas less polar solvents or sequential partitioning are required to efficiently recover triterpenoids and polyacetylenes (Table 2). Without harmonized protocols linking solvent polarity and extraction conditions to specific compound classes, meaningful cross-study comparisons remain difficult.

This challenge is further compounded by heterogeneity in chromatographic analysis. Although reverse-phase C18 columns dominate DM phytochemical profiling, variations in mobile-phase composition, gradient programs, and detection strategies frequently lead to inconsistent retention behavior and limited reproducibility. These issues underscore the need for validated, community-adopted analytical workflows with clearly defined parameters for extraction, separation, detection, and quantification. The standardized HPLC conditions (Table 3) proposed in this review provide a practical foundation for improving inter-laboratory consistency and enabling more reliable correlations between chemical profiles and biological outcomes.

**Figure 3:**
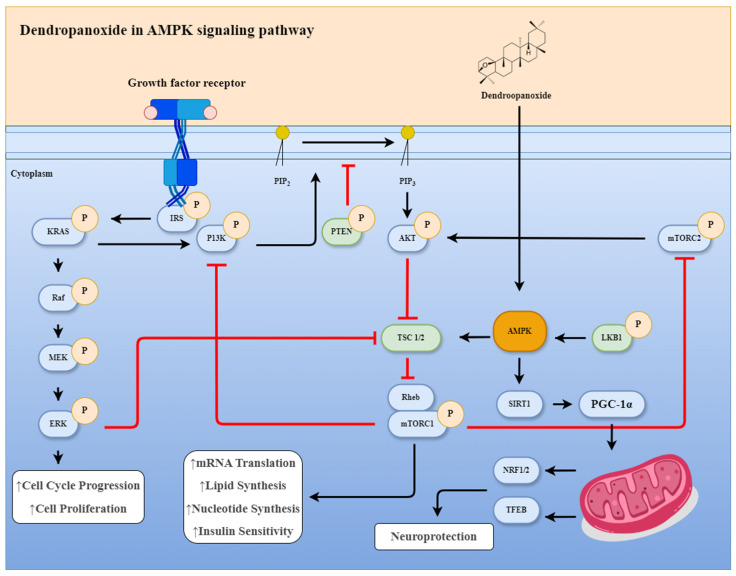
Proposed mechanism of action of dendropanoxide in metabolic and neurodegenerative disease models. Based on its diterpenoid physicochemical properties, dendropanoxide is predicted to be taken up by cells via a specific transporter. Once internalized, dendropanoxide activates AMP-activated protein kinase (AMPK), leading to inhibition of mammalian target of rapamycin complex 1 (mTORC1) signaling, suppression of lipogenesis, and improvement of insulin sensitivity. Concurrently, AMPK activation stimulates peroxisome proliferator-activated receptor gamma coactivator 1-alpha (PGC-1α), promoting mitochondrial biogenesis. Downstream targets of PGC-1α include nuclear respiratory factor 1/nuclear factor erythroid 2-related factor 2 (NRF1/2) and transcription factor EB (TFEB), which enhance mitochondrial function and autophagy/lysosomal biogenesis, respectively, thereby contributing to neuroprotective effects. In this signaling scheme, black arrows denote activation or the stimulation of downstream targets, whereas red T-bars represent inhibitory interactions between pathway components.

Beyond analytical considerations, mechanistic validation of DM-derived bioactive compounds remains incomplete. Dendropanoxide, a diterpenoid unique to DM, exemplifies this gap. Existing studies suggest that it influences key signaling pathways involved in energy metabolism [8,55], inflammation [29], and neuronal homeostasis [11], including AMPK, nuclear factor kappa B (NF-κB), and antioxidant response pathways. Figure 3 presents a hypothetical and integrative model based on currently available preclinical evidence, and is intended to provide a conceptual framework rather than a definitive mechanistic pathway. These findings highlight the therapeutic promise of dendropanoxide and related constituents.

Nevertheless, most mechanistic insights are derived from indirect cellular readouts [30,31,97] or animal models [8,29,55], and direct evidence of molecular target engagement remains scarce. Critical questions—such as whether dendropanoxide directly binds AMPK or modulates it indirectly through upstream regulators—have yet to be resolved. Addressing these uncertainties will require systematic application of biophysical binding assays and orthogonal validation strategies to distinguish direct molecular interactions from downstream or compensatory effects.

An additional conceptual limitation of current DM research is its predominant focus on individual compounds. As a chemically complex botanical, DM is likely to exert its biological effects through the combined action of multiple constituents rather than through a single dominant molecule. Phenolic acids and flavonoids may contribute antioxidant and cytoprotective effects, while diterpenoids and triterpenoids may modulate metabolic and inflammatory signaling, potentially resulting in additive or synergistic outcomes.

Future studies would benefit from integrating network pharmacology, multi-omics analyses, and experimentally validated combination studies to capture these interaction effects. Such approaches are also essential for evaluating potential herb–drug interactions, particularly in chronic disease contexts where DM-derived products may be used alongside conventional pharmacotherapies.

Despite encouraging biological data, translational progress is constrained by a notable lack of pharmacokinetic and exposure information. For most DM-derived compounds, including dendropanoxide, data on bioavailability, metabolic stability, tissue distribution, and achievable systemic concentrations are extremely limited. This gap makes it difficult to assess whether concentrations used in in vitro studies are physiologically relevant or attainable in vivo.

Systematic pharmacokinetic and toxicological studies are therefore essential to bridge the divide between mechanistic promise and practical application. Such data will be particularly critical for advancing DM constituents toward functional food standardization or therapeutic development.

In summary, advancing DM research will require an integrated strategy that aligns standardized analytical methodologies with rigorous mechanistic validation and translationally relevant pharmacokinetic assessment. Establishing harmonized extraction and chromatographic protocols will improve reproducibility, while deeper molecular interrogation of key compounds such as dendropanoxide will clarify their true modes of action. Finally, embracing multi-component perspectives and exposure-driven evaluation frameworks will be essential for translating DM’s chemical diversity into reliable and effective applications in nutrition and medicine.

## 7. Conclusions

Research on *Dendropanax morbifera* (DM) has advanced significantly, with numerous studies demonstrating its broad spectrum of health benefits. DM contains various compounds, including phenolic acids, flavonoids, triterpenoids, diterpenoids such as dendropanoxide, and polyacetylenes, each contributing distinct biological effects. These constituents exhibit antioxidant, anti-inflammatory, antidiabetic, neuroprotective, hepatoprotective, and other health-promoting activities. These findings support DM’s traditional uses and highlight its potential for novel therapeutic and health product development [1,2,6,18,20,94,97,98,99,100,101,102,103,104,105,106,107,108,109,110,111].

Despite these advances, key challenges remain before DM research can be fully standardized and translated into practice. The primary concern is the need for rigorous standardization of laboratory methods, particularly for extraction and HPLC analysis. Variations in solvents, extraction procedures, and chromatographic conditions have led to inconsistent results, complicating cross-study comparisons. The protocols proposed in this review—including appropriate solvent selection, plant-part-specific extraction methods, and standardized HPLC conditions—provide a practical framework to enhance consistency and comparability. Implementing these methods will minimize variability and offer a clearer understanding of DM’s chemical and biological properties.

Mapping interactions between DM compounds and their molecular targets is essential for linking the plant’s chemistry to its biological effects. Several DM constituents interact with key proteins, including sirtuins (SIRT1, SIRT6), AMP-activated protein kinase (AMPK), cannabinoid receptors, the pregnane X receptor (PXR), and cytochrome P450 enzymes. Dendropanoxide, a diterpenoid unique to DM, has shown preliminary evidence of activating AMPK and influencing glucose metabolism, lipid breakdown, and neuroprotection. While these findings are promising, further validation through binding studies, structure–activity relationship (SAR) analysis, and animal experiments is necessary to confirm their mechanisms and therapeutic potential.

This review also incorporates in silico analyses using SwissTargetPrediction (STP) to identify potential targets of DM compounds. Predicted targets include kinases, oxidoreductases, carbonic anhydrases, GPCRs, proteases, and nuclear receptors, which are involved in inflammation, stress response, metabolism, and neuroprotection. Targets such as AKT1, GSK3B, MMP9, BACE1, CYP1B1, and protein tyrosine phosphatase, receptor type S (PTPRS) align with previous DM research, indicating that DM compounds may modulate multiple biological pathways simultaneously. To assess the reliability of these computational predictions, the study compared them with known targets of well-characterized compounds. SwissTargetPrediction (STP) matched approximately 70–100% of the targets for quercetin, kaempferol, resveratrol, luteolin, ferulic acid, and cannabidiol. This demonstrates the tool’s value for identifying novel targets and guiding early-stage research.

Future DM research should prioritize investigating the combined effects of its multiple compounds rather than focusing solely on individual constituents. The health benefits of DM may result from synergistic or complementary interactions among its diverse chemicals. Employing network pharmacology and multi-omics approaches, such as metabolomics, transcriptomics, and proteomics, can help identify cooperative compound groups, reveal novel mechanisms of action, and assess potential risks when used alongside other drugs. Specific evaluation methods, such as combination index analysis and computational network analysis, should be suggested to enable researchers to effectively design studies that explore potential synergies in multi-compound effects [112]. Advancing DM research will also require reproducibility across laboratories and improved data sharing. A clear research framework—including standardized HPLC analysis, mapping compound–protein interactions, validating mechanisms of action, and investigating compound synergy—will guide future studies. Collaborative efforts are essential to establish DM as a well-characterized resource for medicine, nutrition, and cosmetics.

In conclusion, this review integrates current knowledge on the chemistry, bioactivities, analytical methodologies, and molecular target interactions of *Dendropanax morbifera*. While substantial progress has been made, further advancement of the field will depend on the adoption of standardized extraction and chromatographic protocols, rigorous mechanistic validation of key constituents, and improved consideration of pharmacokinetics and exposure. Importantly, future research should move beyond a reductionist focus on individual compounds and instead embrace the intrinsic chemical complexity of DM. Taken together, the available evidence positions *Dendropanax morbifera* not merely as a source of isolated bioactive molecules, but as a systems-level phytochemical resource in which multi-component interactions, network-level target engagement, and contextual synergy collectively define its biological and translational potential.

## Figures and Tables

**Table 1:** Summary of representative bioactive compounds isolated from *Dendropanax morbifera* and their experimentally reported biological activities.

Bioactivity	Compound	Experimental Model	Reference
**Anti-cancer**	3,5-Dicaffeolyquinic acid	In vitro (A549 cell)	[40]
	Dihydroconiferyl Ferulate	In vitro (MDA-MB-231 cell, MCF-7 cell)	[52,53]
	Rosmarinic acid	In vitro (Huh-7 cell)	[54]
**Antioxidation**	Schaftoside	In vitro (DPPH assay)	[36]
	Syringin		
	6-Hydroxyluteolin 7-O-laminaribioside		
	Kaempferol-3-O-rutinoside	In vitro (DPPH assay)	[16]
	cis-6-Oxogeran-4-enyl-10-oxy-O-β-arabinopyranosyl-4′-O-β-arabinopyranosyl-2″-octadec-9″′,12″′,15″′-trienoate	In vitro (DPPH assay)	[7]
	Geran-3(10)-enyl-1-oxy-O-β-arabinopyranosyl-4′-O-β-arabinopyranosyl-2″-octadec-9″′,12″′,15″′-trienoate		
	Caffeic acid	In vitro (DPPH assay)	[35]
	Isoquercitrin	In vitro (HT22 cell)	[19]
	Quercetin	In vitro (HaCaT keratinocytes)	
**Anti-inflammatory**	3,5-dicaffeolyquinic acid	In vitro (RAW 264.7 cell)	[40]
	Dendropanoxide	In vivo (rats)	[55]
	Quercetin	In vitro (RAW 264.7 cell)	[19]
	(+)-Catechin	In vitro (RAW 264.7 cell)	[46]
	Ferulic acid		
	Myricetin		
	α-Amyrin	In vitro (RAW 264.7 cell)	[56]
	β-Amyrin		
**Anticomplement**	(3S)-Falcarinol	In vitro (Complement pathway assay)	[41]
	(3S,8S)-Falcarindiol		
	(3S)-Diynene		
**Cognitive Enhancement**	Orientin	In vivo (mouse)	[16]
	Isoorientin		
	Luteolin-7-O-rutinoside		
**Antidiabetic**	Dendropanoxide	In vivo (rats)	[8]
**Neuroprotective**	Quercetin	In vitro (HT22 cell)	[57]
	Isoquercitrin		
	Syringin	In vivo (mouse)	[49]
**Anti-fibrotic**	Syringin	In vivo (rats)	[18]
**Hepatoprotective**	(9Z,16S)-16-hydroxy-9,17-octadecadiene-12,14-diynoic acid	In vitro (HepG2 cell)	[58]
**Nephroprotective**	Dendropanoxide	In vitro (NRK-52E cell)	[55]

**Table 2:** Proposed standardized extraction conditions for *Dendropanax morbifera*. The table summarizes recommended solvents, extraction methods, and plant parts for the efficient isolation of major phytochemical classes, with the aim of improving reproducibility, optimizing compound-specific recovery, and facilitating cross-study comparability.

Phytochemical Class	Solvent	Method	Plant Part	Key Notes
Phenolic acids (e.g., chlorogenic acid, caffeic acid)	70–80% EtOH	24 h shaking; vacuum concentration	Leaves	Major antioxidant constituents
Flavonoids (e.g., rutin, quercetin, kaempferol)	80% EtOH	Ultrasonic extraction (30 min); filtration	Leaves, stems	Anti-inflammatory and immunomodulatory activities
Diterpenoids/Triterpenoids (e.g., dendropanoxide, α-/β-amyrin)	80% MeOH	Ultrasonic or reflux extraction	Bark, roots	MS detection due to low UV absorbance
Polyacetylenes (falcarinol derivatives)	80% MeOH → liquid–liquid partitioning (hexane, CHCl_3_, ethyl acetate (EtOAc))	Maceration (48 h); vacuum concentration	Bark	Charged aerosol detector (CAD) or MS detection
Water-soluble compounds (e.g., syringin)	Water	Hot-water extraction (100 °C, 2 h)	Leaves, stems	Suitable for food/pharmaceutical applications

The arrow (**→**) represents a procedural transition.

**Table 3:** Recommended HPLC conditions used for the analysis of major compound groups from Dendropanax morbifera.

Phytochemical Class	Representative Compounds	Mobile Phase	Gradient Program	Detection λ (nm)	Notes
**Phenolic acids**	Chlorogenic acid, caffeic acid, ferulic acid, etc.	H_2_O (0.1% FA)/ACN	10–80% ACN over 30–40 min	320–330	Validated for chlorogenic and caffeic acids
**Flavonoids**	Rutin, quercetin, kaempferol, etc.	H_2_O (0.1% FA)/ACN	10–80% ACN over 30–40 min	254–280	Suitable for rutin and quercetin; UV or photodiode array (PDA)
**Diterpenoids** **Triterpenoids**	Dendropanoxide, α-amyrin, β-amyrin, friedelin, β-sitosterol	H_2_O (0.1% FA)/ACN	20–90% ACN over 40 min	210–254	Dendropanoxide typically detected at 254 nm; many diterpenoids have weak UV absorbance
**Polyacetylenes**	(3S)-Falcarinol, (3S,8S)-falcarindiol, (3S)-diynene, (9Z,16S)-16-hydroxy-9,17-octadecadiene-12,14-diynoic acid	H_2_O (0.1% FA)/MeOH	30–90% MeOH over 40 min	220	CAD or MS often used for confirmation due to low UV sensitivity
**Others**	Syringin, saponins, rosmarinic acid	H_2_O (0.1% FA)/ACN	10–80% CAN over 30 min	Variable (UV or MS)	Syringin and saponins frequently require MS detection for adequate sensitivity.

## Data Availability

The data supporting this article are included as Supplementary Materials.

## Supplementary Materials

The following supporting information can be downloaded at https://www.mdpi.com/article/10.3390/life16010100/s1. Table S1: Detailed list of compounds identified from *Dendropanax morbifera* across different extraction solvents.; Table S2: Summary of extraction methods, plant parts, identified compounds, and analytical detection techniques reported for *Dendropanax morbifera*; Table S3: Detailed HPLC analytical conditions, including mobile phase compositions; Table S4: Experimental models, experimental conditions, key mechanisms, and bioactivities of rutin and chlorogenic acid; Table S5: Key bioactive compounds isolated from *Dendropanax morbifera* and their reported biological activities and mechanisms of action. SwissTargetPrediction results are presented in the file STP results.xls. References [113,114,115] are cited in the supplementary materials.
