# Supplementary material for: Unifying Phytochemistry, Analytics, and Target Prediction to Advance Dendropanax morbifera Bioactive Discovery
Source: Life (Basel). 2026 Jan 11;16(1):100. doi: 10.3390/life16010100 (PMC12843060; doi:10.3390/life16010100)
Supplement: Supplementary file 1 [file life-16-00100-s001.zip › Supplementary tables.pdf]

Supplementary Tables

Supplementary Table S1. Detailed list of compounds identified from *Dendropanax morbifera* across different extraction solvents

Extraction Method	Plant Part Used	Compound Identified	Detection Method	Reference
70% EtOH, ultrasound-assisted extraction (80°C, 1 hr)	Root	3,5-DCQA (3,5-dicaffeoylquinic acid)	Electrospray Ionization	[1]
80% EtOH extraction followed by hexane, chloroform, and ethyl acetate fractionation	Leaves	Rutin, Orientin, Isoorientin, Luteolin-7-O-rutinoside	UPLC-QTOF/MS	[2]
MeOH extraction	Leaves	(3S)-Falcarinol, (3S,8S)-Falcarindiol, (3S)-Diynene	PDA Detector	[3]
MeOH extraction (reflux at 30°C, 4 hrs, repeated three times)	Leaves	Dendropanoxide	UV Detection	[4]
Hot-water and EtOH extraction (30%, 50%, 70%) at 80°C for 2 hrs	Leaves	Chlorogenic acid, Rutin	LC-MS/MS	[5]
MeOH extraction (ultrasonic extraction at 55°C for 1 hr, repeated three times)	Leaves	Rutin, Chlorogenic acid, Quercetin, (+)-Catechin, Ferulic acid, Myricetin, p-Coumaric acid, p-Hydroxybenzoic acid, Naringenin, Protocatechuic acid, Kaempferol, Vanillin, m-Coumaric acid, trans-Cinnamic acid, Syringic acid, Naringin, Gallic acid, o-Coumaric acid, Hesperetin, Biochanin A, Resveratrol, Formononetin	UV Detection	[6]
Boiling EtOH extraction (reflux, three times)	Leaves, Stem	((10E)-9,16-Dihydroxyoctadeca-10,17-dien-12,14-diynoate, (10E)-(–)-10,17-Octadecadiene-12,14-diyne-1,9,16-triol	LC-MS/MS	[7]

30% & 60% EtOH (70–75°C, 3–4 hrs)	Leaves	Chlorogenic acid, Rutin	Electrospray Ionization	[8]
Hot-water extraction	Leaves	Chlorogenic acid, Rutin	UV Detection	[9]
Hot-water extraction (100°C, 2 hrs)	Leaves	Chlorogenic acid, Rutin, Quercetin, Kaempferol, Vitexin, Luteolin, Tricin, Ferulic acid, Caffeic acid	UV Detection	[10]
Hot-water extraction (80°C for 2 or 4 hrs); fermented EtOH extraction (30–95%) at 80°C	Leaves, Stem, Root	Chlorogenic acid, Rutin	Diode-Array Detector	[11]
Hot-water extraction (85°C, 3 hrs); 30% EtOH extraction (75°C, 4 hrs); 60% EtOH extraction (70°C, 4 hrs)	Leaves	α-Amyrin, β-Amyrin, Chlorogenic acid, 4-Methylcatechol, p-Coumaric acid, Rutin	Triple TOF	[12]
70% EtOH or water extraction at 80°C for 3 hrs (repeated three times)	Leaves, Stem	Rutin, Taxifolin, Neohesperidin, Hesperidin, Caffeic acid, Chlorogenic acid	PDA Detector	[13]
70% EtOH (1:10 sample-to-solvent, 80°C, 14 hrs)	Leaves	Chlorogenic acid, Rutin	Diode-Array detector	[14]
Water extraction at 90°C for 5 hrs, concentrated under reduced pressure	Leaves, Stem	Neochlorogenic acid, Syringin, Chlorogenic acid, Rutin	UV Detection	[15]
EtOH extraction (soaking powder in EtOH for 1 week at room temp, centrifugation, evaporation at 70°C)	Leaves	Quercetin, Chlorogenic acid, Rutin, Carnosol, Cannabidiol, Dextromethorphan, Bremazocine, Doxapram, Resolvin D2, Procyclidine, 2-Arachidonoylglycerol, Eplerenone	LC-MS/MS	[16]
Hot-water extraction (100°C, 4 hrs) and EtOH extraction (20–100%) for 3 days at room temp	Leaves	Chlorogenic acid, Rutin	UV Detection	[17]

MeOH extraction followed by ethyl acetate extraction	Leaves	Dihydroconiferyl ferulate	UV Detection	[18]
Soxhlet extraction; ultrasound-assisted extraction (UAE)	Leaves	Neochlorogenic acid, Chlorogenic acid, Cryptochlorogenic acid, Rutin	UV Detection	[19]
70% EtOH reflux extraction (80°C, 2 hrs)	Leaves	Gallic acid, 3,4-Dihydroxybenzoic acid, Rutin, Chlorogenic acid, Caffeic acid, p-Coumaric acid, trans-Ferulic acid, Quercetin	UV Detection	[20]
Water extraction followed by fractionation (n-hexane, chloroform, ethyl acetate, n-butanol, water)	Leaves	Rutin	UV Detection	[21]
MeOH extraction (55°C ultrasound, evaporation, lyophilization)	Leaves, Debarked stem, Bark, Branch	Caffeic acid, Chlorogenic acid, Rosmarinic acid, Rutin	Diode-Array Detector	[22]
MeOH extraction followed by partition with hexane and ethyl acetate	Leaves, Stem	Syringin, Hyperoside, Koaburside, Epifriedelanol	UV Detection	[23]
Aqueous extraction (100°C, 4 hrs, freeze-dried)	Leaves	Neochlorogenic acid, Chlorogenic acid, Cryptochlorogenic acid, Quercetin, Kaempferol	Diode-Array Detector	[24]
Hot-water extraction (80°C)	Leaves	Syringin, 6-Hydroxyluteolin 7-O-laminaribioside, Schaftoside, Rutin, Kaempferol-3-O-rutinoside	UV Detection	[25]
80% EtOH extraction with reflux for 2 hrs	Leaves	Rutin, Chlorogenic acid, (+)-Catechin, Ferulic acid, Quercetin, Myricetin	UV Detection	[26]
Microwave-assisted extraction	Leaves	Quinic acid, 1-O-Caffeoylquinic acid, 4-O-Caffeoylquinic acid, Apocynoside I, Viscumneoside III, Quercetin	UV Detection	[27]

Acetone extraction (3 × 2.5 L, room temp, 3 days)	Bark	cis-6-Oxogeran-4-enyl-10-oxy-O-β-arabinopyranosyl-40-O-β-arabinopyranosyl-2″-octadec-9″′,12″′,15″′-trienoate, geran-3(10)-enyl-1-oxy-O-β-arabinopyranosyl-40-O-β-arabinopyranosyl-2″-octadec-9″′,12″′,15″′-trienoate, Geranilan-8-oxy-O-α-d-xylopyranosyl-20-n-octadec-9″,12″,15″-trienoate 1-cyclohex-20,50-dienyl 1-cyclohexylethanol-O-β-d-xylopyranoside, Guaiacol-O-β-d-arabinopyaranoside n-tetradecanyl oleate, Oleyl-O-β-d-xyloside, n-octadec-9,12-dienoyl-O-β-d-arabinopyranoside, Linolenyl-O-β-d-arabinofuranoside, Glyceryl-1, 3-dipalmito-2-olein	UV Detection	[28]
MeOH extraction (41.23% v/v, 88.61°C, 1.86 hrs)	Leaves	Caffeic acid	UV Detection	[29]
95% EtOH extraction with ultrasound (40 kHz, 300 W, 15-min cycles)	Leaves	(9Z,16S)-16-Hydroxy-9,17-octadecadiene-12,14-diynoic acid	Charged Aerosol Detector	[30]
Sepbox 2D-5000 fractionation (RP C18 column)	Leaves	(9Z,16S)-16-Hydroxy-9,17-octadecadiene-12,14-diynoic acid	UV Detection	[31]
EtOH extraction (80% EtOH, 24-hr shaking) + MeOH extraction + hot-water extraction + chloroform maceration	Bark, Leaves, Root	Dendropanoxide, Chlorogenic acid, Caffeic acid, Saponins	UV Detection	[32]
70% MeOH reflux followed by liquid-liquid extraction	Leaves	Chlorogenic acid, Rutin, Hyperoside, Isoquercitrin, Quercetin	UV Detection	[33]
Water extraction at 121°C for 6 hrs	Leaves, Stem	Rutin, Vitexin, Syringin, Chlorogenic acid, Neochlorogenic acid	UV Detection	[34]

Supplementary Table S2. Summary of extraction methods, plant parts, identified compounds, and analytical detection techniques reported for *Dendropanax morbifera*.

Category	Summary (PRISMA-style condensed form)
**Total studies included**	*n* = 34
**Plant parts reported**	Leaves (dominant), Stem, Bark, Root, Branch
**Major extraction solvents**	Water (hot-water), EtOH (20–95%), MeOH, Acetone, Mixed solvent fractionation (Hexane, $CHCl_3$, EtOAc, n-BuOH)
**Extraction methods**	Hot-water extraction, Reflux extraction, Ultrasonic-assisted extraction (UAE), Microwave-assisted extraction (MAE), Soxhlet extraction, Maceration, Fermented-EtOH extraction, Liquid–liquid partitioning
**Temperature range**	25°C (room temperature maceration) → 121°C (pressurized water extraction)
**Extraction duration**	1–14 hours (typical) → up to 1 week (EtOH soaking)
**Detection platforms**	UV Detection, PDA/Diode-array detector, LC-MS/MS, UPLC-QTOF/MS, ESI-MS, TOF-MS, Charged Aerosol Detector
**Compound classes identified**	Phenolic acids, Flavonoids, Terpenoids (diterpenoids/triterpenoids), Polyacetylenes, Lignans, Glycosides, Long-chain esters
**Most frequently identified compounds**	Chlorogenic acid, Rutin (most frequent), Quercetin, Kaempferol, Caffeic acid, Neochlorogenic acid
**Solvent–compound specificity**	
→ **Water**	Chlorogenic acid, Rutin, Syringin, Neochlorogenic acid, Vitexin
→ **EtOH (30–95%)**	Chlorogenic acid, Rutin, Quercetin, Catechin, Ferulic acid, Myricetin
→ **MeOH**	Dendropanoxide, Falcarinol derivatives, Polyacetylenes, Terpenoids
→ **Acetone**	Long-chain glycosides and fatty acyl esters (bark-specific)
→ **Fractionation (Hexane/$CHCl_3$/EtOAc)**	Polyacetylenes, Luteolin glycosides, Syringin, Hyperoside
**Notable unique compounds**	Dendropanoxide (diterpenoid), Falcarinol & falcarindiol derivatives (polyacetylenes), (9Z,16S)-16-hydroxy-9,17-octadecadiene-12,14-diynoic acid
**Part–compound association patterns**	
Leaves	Highest diversity: phenolics, flavonoids, polyacetylenes
Stem	Syringin, Rutin, Caffeic derivatives
Bark	Dendropanoxide, long-chain glycosides, terpenoids
Root	3,5-DCQA, limited but distinct caffeoylquinic acids
**Analytical reliability**	LC-MS/MS & UPLC-QTOF/MS (high-confidence), UV/PDA detection (commonly used but semi-quantitative)
**Method trends**	Hot-water extraction consistently detects chlorogenic acid + rutin; Alcohol extraction increases detection of flavonoids & phenolic acids; MeOH strongly associated with terpenoids and polyacetylenes

<PRISMA Narrative Summary of Extraction Evidence>

A total of 34 studies met the eligibility criteria and were included in the extraction analysis for *Dendropanax morbifera*. Most studies utilized leaves as the primary plant material, followed by stem, bark, and root. Extraction conditions varied widely, with water, ethanol (20–95%), and methanol being the predominant solvents. Common extraction procedures included hot-water extraction, ultrasound-assisted extraction (UAE), reflux extraction, maceration, microwave-assisted extraction, and solvent fractionation.

Across studies, more than 40 unique phytochemicals were reported, mainly belonging to phenolic acids, flavonoids, polyacetylenes, terpenoids, lignans, and glycosides. Among these, chlorogenic acid and rutin were the most consistently detected compounds across different extraction conditions and plant parts. Hot-water extraction reliably yielded chlorogenic acid and rutin, while alcoholic solvents enhanced recovery of flavonoids such as quercetin, kaempferol, myricetin, catechin, and diverse cinnamic acid derivatives. Methanol-based extraction uniquely identified polyacetylenes (e.g., falcarinol derivatives) and diterpenoids such as dendropanoxide, particularly in leaves and bark.

Analytical detection techniques varied, with UV detection being the most frequently employed, although LC-MS/MS, UPLC-QTOF/MS, and ESI-MS provided higher-confidence identification. Fractionation using hexane, chloroform, ethyl acetate, and n-butanol facilitated identification of glycosides and lipophilic constituents including syringin, hyperoside, and long-chain esters.

Overall, extraction outcomes were strongly influenced by solvent polarity, temperature, and plant part, underscoring the need for optimized, standardized extraction protocols for D. morbifera phytochemical profiling.

Supplementary Table S3. Detailed HPLC analytical conditions, including mobile phase compositions

Plant Part	Column	Mobile Phase/Gradient	Detection	Flow Rate (mL/min)	Identified Compounds	Reference
Leaves	ACQUITY UPLC BEH C18 (2.1 × 100 mm, 1.7 μm)	A: 0.1% formic acid in water; B: acetonitrile, 0–0.5 min: 0% B → 0.5–8 min: 0–100% B → 8–8.5 min: 100% B → 8.5–10 min: 100–0% B → 10–11 min: 0% B	UPLC-QTOF/MS[2]	0.4	Rutin, Orientin, Isoorientin, Luteolin-7-O-rutinoside	[2]
	Rainin Dynamax Cyano (4.1 × 250 mm)	Isocratic: Hexane:Isopropanol (45:5, v/v)	PDA 258 nm	50	(3S)-Falcarinol, (3S,8S)-Falcarindiol, (3S)-Diynene	[3]
	YMC-Pack Pro C18 (250 × 20 mm, 5 μm)	Acetonitrile:Water (30:70, v/v), isocratic	UV 254 nm	5.0	Dendropanoxide	[4]
	Kinetex C18 (150 × 2.1 mm, 2.6 μm)	A: 5 mM ammonium acetate + 0.1% FA in water; B: MeOH + 2.5 mM ammonium acetate, 0–8 min: 90→80% B → 8–30 min: 80→55% B → 30–60 min: 55→30% B	LC-MS/MS	0.3	Chlorogenic acid, Rutin	[5]
	Thermo Accucore C18 (2.1 × 100 mm, 2.6 μm)	A: 0.1% acetic acid in water; B: 0.1% acetic acid in ACN. Gradient: 98% A → 15 steps to 100% B → return to 98% A	UV 280 nm	0.5	Rutin, Chlorogenic acid, Quercetin, Catechin, Ferulic acid, Myricetin, p-Coumaric acid, Naringenin, Protocatechuic acid, Kaempferol, Vanillin, etc.	[6]
	Eclipse Plus C18 (4.6 × 250 mm, 5 μm)	A: 0.1% FA in water; B: 0.1% FA in ACN. Linear 0–56 min (0 → 100% B)	UV 310 nm	1.0	Chlorogenic acid, Rutin	[8]
	C18 (2.1 × 100 mm, 2.7 μm)	A: 0.1% FA in water; B: 0.1% FA in ACN; 95→50% A (0–40 min)	HPLC-ESI-MS/MS	0.4	Chlorogenic acid, Rutin, Quercetin, Kaempferol, Vitexin, Luteolin, Tricin, Ferulic acid, Caffeic acid	[10]
	Halo C8 (2.0 × 75 mm) and Capcell Pak C18 MG	Separate gradients for amyrins / polyphenols	TOF-MS, UV 280 nm	0.4 / 1.0	α-Amyrin, β-Amyrin, Chlorogenic acid, Rutin, etc.	[12]
	YMC Triart C18	TFA/Water & TFA/ACN gradient system	DAD (260/310/365 nm)	1.0	Gallic acid, Rutin, Chlorogenic acid, Caffeic	[14]

(250 × 4.6 mm)				acid, Ferulic acid, Quercetin, Kaempferol	
Accucore C18 (2.1 × 150 mm, 3 µm)	A: Water/ACN (60:40) + 10 mM ammonium formate + 0.1% FA; B: IPA/ACN (90:10) + ammonium formate; multi-step gradient 0–18 min	LC–MS/MS (Q-Exactive, ESI+)	0.2	Quercetin, Chlorogenic acid, Rutin, Carnosol, Cannabidiol, Dextromethorphan, Bremazocine, Doxapram, Resolvin D2, Procyclidine, 2-AG, Eplerenone	[16]
Zorbax Extended-C18 (4.6 × 150 mm, 5 µm)	A: ACN; B: 0.2% phosphoric acid; 10→25% A (0–20 min), 100% A (21 min)	UV 330 nm	0.8	Chlorogenic acid, Rutin	[17]
ODS (10 × 250 mm)	Water/ACN; 0%→100% B (0–30 min), hold	UV 220 nm	2.0	Dihydroconiferyl ferulate	[18]
ZORBAX Eclipse Plus C18 (250 × 4.6 mm, 5 µm)	A: 0.4% phosphoric acid; B: ACN; 5→30% B (0–60 min)	UV 327 nm	1.0	Neochlorogenic acid, Chlorogenic acid, Cryptochlorogenic acid, Rutin	[19]
YMC Triart C18 (250 × 4.6 mm, 5 µm)	A: 0.2% FA in water; B: ACN; multiple shallow gradients	UV 260/310/365 nm	0.8	Gallic acid, 3,4-DHBA, Rutin, Chlorogenic acid, Caffeic acid, p-Coumaric acid, Ferulic acid, Quercetin	[20]
Shim-pack VP-ODS (150 × 4.6 mm)	A: 0.1% FA in water; B: MeOH; 5→100% B (0–12 min)	UV 360 nm	1.0	Rutin	[21]
Eclipse XDB-C18 (4.6 × 250 mm, 5 µm)	Organic acids: 10→0% A (0–25 min); Flavonoids: TFA/water–ACN gradient	DAD 370 nm	1.0	Neochlorogenic acid, Chlorogenic acid, Cryptochlorogenic acid, Quercetin, Kaempferol	[24]
ProntoSIL C18 (250 × 4.6 mm, 5 µm)	A: Water; B: 0.1% FA in ACN; 10→95% B (0–70 min)	UV 280 nm	0.5	Syringin, 6-Hydroxyluteolin 7-O-laminaribioside, Schaftoside, Rutin, Kaempferol-3-O-rutinoside	[25]
Accucore C18 (2.1 × 100 mm, 2.6 µm)	0.1% Acetic acid (A/B) gradient (0→45% B, 0–14 min)	UV 280 nm	0.5	Rutin, Chlorogenic acid, Catechin, Ferulic acid, Quercetin, Myricetin	[26]

	ACQUITY UPLC HSS T3 (1.8 μm)	FA/water – ACN; 97% A → 100% B (1–15 min)	UV 265 nm	0.5	Quinic acid, 1-O-CQA, 4-O-CQA, Apocynoside I, Viscumneoside III, Quercetin	[27]
	Symmetry C18 (3.9 × 150 mm, 5 μm)	0.5% Acetic acid/MeOH (isocratic 50:50)	UV 330 nm	1.0	Caffeic acid	[29]
	Grace Davisil C18 (50 × 500 mm, 10 μm)	Water–MeOH; 10→100% B (0–180 min)	UV 254 nm, ELSD	30	(9Z,16S)-16-Hydroxy-9,17-octadecadiene-12,14-diynoic acid	[30]
	Waters XBridge C18 (4.6 × 150 mm, 5 μm)	Water–ACN; 30→70% B (0–20 min)	UV 254nm	1.0	Rutin, Quercetin	
	Zorbax Eclipse Plus C18 (3.0 × 100 mm, 3.5 μm)	Water–MeOH (isocratic 60% B)	DAD 280/320/360 nm	0.8	Catechins, Epicatechin gallate	[35]
	Phenyl-Hexyl (Luna, 2.1 × 100 mm, 2.6 μm)	0.1% FA in water/ACN; 20→ 80% B	FLD (Ex 280/Em 450)	1.2	Kaempferol, Myricetin	
	Hypersil GOLD C18 (250 × 4.6 mm)	0.1% FA/water–ACN; 5→ 100% B (0–25 min)	UV 254 nm	0.8	Chlorogenic acid, Rutin, Hyperoside, Isoquercitrin, Quercetin	[33]
Leaves, Stem	Gemini C18 (50 × 2.0 mm)	0.1% FA in water/ACN; 15→ 60% B	LC-MS/MS (Q-Trap)	0.3	(10E)-9,16-Dihydroxyoctadeca-10,17-dien-12,14-diynoate, (10E)-(–)-10,17-Octadecadiene-12,14-diyne-1,9,16-triol	[7]
	Triart C18 (250 × 4.6 mm)	Long-step gradients for flavonoids and phenolic acids	PDA 280/245 nm	0.8–1.0	Rutin, Taxifolin, Neohesperidin, Hesperidin, Caffeic acid, Chlorogenic acid	[13]
	Kinetex C18 (150 × 4.6 mm, 5 μm))	10→100% MeOH (45 min)	UV 254 nm	1.0	Neochlorogenic acid, Syringin, Chlorogenic acid, Rutin	[15]
	YMC-Actus Triart C18	Water + 0.1% FA / ACN (20 →80% B, 30 min)	DAD 254/280/320 nm	1.0	Falcarinol, Falcarindiol, Syringin, Hyperoside, Koaburside,	[23]

	(250 × 4.6 mm, 5 μm)				Epifriedelanol, Dihydroconiferyl ferulate	
	Luna C18 (4.6 × 250 mm, 5 μm)	0.1% FA/water–ACN; 95:5 → 60:40	UV 250 nm	1.0	Rutin, Vitexin, Syringin, Chlorogenic acid, Neochlorogenic acid	[34]
Bark	Shim-pack XR-ODS (150 × 2.0 mm, 2.2 μm)	0.1% TFA in water–ACN; 20 →80% B	UV 254/DAD 280–320 nm)	0.5	Multiple glycosylated fatty acid esters	[28]
Bark. Leaves, Root	Waters SunFire C18 (150 × 4.6 mm, 5 μm)	Water + 0.1% FA / ACN; 10 →90% B	DAD 220/254/280 nm	1.0	Dendropanoxide, Quercetin, Kaempferol, Rutin, Gallic acid, Caffeic acid	[32]
Leaves, Debarked stem, Bark, Branch	Poroshell 120 EC-C18 (4.6 × 100 mm, 2.7 μm)	MeOH / 2% AcOH; 0→50% A (0–15 min)	DAD 320/370 nm	1.0	Caffeic acid, Chlorogenic acid, Rosmarinic acid, Rutin	[22]
Leaves, Stem, Root	Eclipse Plus C18 (4.6 × 250 mm, 5 μm)	0.1% FA in water–ACN; 100 →40% A (0–20 min)	DAD 310 nm	1.0	Chlorogenic acid, Rutin	[11]
Adventitious root	ZORBAX Eclipse Plus C18 (250 × 4.6 mm, 5 μm)	0.1% AcOH/water–MeOH; 90→30% B	ESI negative	1 (HPLC), 0.3 (LC-MS)	3,5-Dicaffeoylquinic acid	[1]

Supplementary Table S4. Experimental models, experimental conditions, key mechanisms, and bioactivities of rutin and chlorogenic acid

Compound	Experimental Model	Experimental Condition/Identification	Key Mechanism	Bioactivity	Reference
**Rutin**	In vivo (HFD-induced diabetic mice)	Identified via UPLC–QTOF/MS	Neuroprotection; antioxidant modulation	Cognitive enhancement	[2]
**Chlorogenic acid, Rutin**	In vitro (Xanthine oxidase inhibitory assay)	Dose-dependent XO inhibition	Antioxidant; xanthine oxidase inhibition	Antihyperuricemic activity	[5]
**Rutin, Chlorogenic acid**	In vitro (RAW 264.7 macrophages)	Identified via HPLC	Antioxidant; suppression of pro-inflammatory mediators	Anti-inflammatory	[6]
**Rutin**	In vitro (ABTS, DPPH, FRAP)	Identified via LC–MS/MS	Radical scavenging; ferric-reducing power	Antioxidant	[8]
**Chlorogenic acid**	In vitro (ABTS, DPPH, FRAP)	Identified via LC–MS/MS	Radical scavenging; ferric-reducing power	Antioxidant	
**Chlorogenic acid**	In vitro (RAW 264.7)	LPS-induced inflammation	iNOS & COX-2 downregulation; ↓ NO and PGE2	Anti-inflammatory	[9]
**Rutin**	In vitro (RAW 264.7)	LPS-induced inflammation	Suppression of TNF-α, IL-6	Anti-inflammatory	
**Chlorogenic acid**	In vitro (BV-2 microglia)	LPS-induced neuroinflammation	NF-κB & JNK inhibition; ↓ iNOS & COX-2	Anti-neuroinflammatory	[10]
**Chlorogenic acid**	In vivo (MPTP-induced Parkinson's mouse model)	200 mg/kg DML	↑ TH expression; ↓ microglial activation (Iba-1)	Neuroprotective; anti-Parkinson's	
**Chlorogenic acid, Rutin**	In vitro (ABTS, DPPH)	HPLC-identified; 70% ethanol extract	Radical scavenging	Antioxidant	[11]

**Chlorogenic acid**	In vitro (RAW 264.7)	LPS stimulation	↓ PGE2; ↓ IL-6, IL-1β, TNF-α	Anti-inflammatory	[12]
**Rutin**	In vitro (RAW 264.7)	LPS stimulation	↓ PGE2; ↓ IL-6, IL-1β, TNF-α	Anti-inflammatory	
**Chlorogenic acid**	In vivo (ethanol-induced liver injury)	100–500 mg/kg for 10 days	↑ SOD, CAT, GST, GR; ↓ AST/ALT	Hepatoprotective antioxidant	
**Rutin**	In vivo (ethanol-induced liver injury)	100–500 mg/kg for 10 days	Gut microbiota modulation (↑ *Bacteroides*, *Allobaculum*)	Hepatoprotective; microbiota regulation	[13]
**Rutin**	In vivo (ethanol-induced liver injury)	100–500 mg/kg	↓ CYP2E1 activity	Hepatoprotective	
**Chlorogenic acid**	In vivo (STZ diabetic rats)	25 mg/kg/day for 28 days	↓ oxidative stress; ↓ IL-1β, IL-6, TGF-β1; ↓ ECM	Anti-fibrotic; nephroprotective	[14]
**Rutin**	In vivo (STZ diabetic rats)	25 mg/kg/day for 28 days	↑ SOD, CAT; ↓ AGE accumulation	Antioxidant; nephroprotective	
**Chlorogenic acid**	In vitro (OPCs)	Identified via LC–MS/MS	↑ Myrf, CNP, PLP expression	Myelin synthesis promotion	[15]
**Rutin**	In vitro (OPCs)	Identified via LC–MS/MS	↑ Axonal contact; ↑ OL membrane area	Oligodendrocyte development	
**Chlorogenic acid, Rutin**	In vitro (DPPH, ABTS, XO assay)	HPLC-UV, 80% ethanolic extract	Radical scavenging; XO inhibition	Antioxidant; antihyperuricemic	
**Chlorogenic acid**	In vitro (3T3-L1 adipocytes)	100 μg/mL	↓ PPARγ, ↓ C/EBPα, ↓ Perilipin	Anti-adipogenic	

**Rutin**	In vitro (3T3-L1 adipocytes)	100 µg/mL	↑ UCP1; ↑ PRDM16	Thermogenic; anti-obesity	[17]
**Chlorogenic acid**	In vivo (pentobarbital sleep model, ICR mice)	100, 200 mg/kg	↑ GABA & serotonin; ↓ MDA; ↑ SOD, CAT, GPx	Sleep promotion; neuroprotection	
**Rutin**	In vivo (pentobarbital sleep model)	100, 200 mg/kg	↑ GABAA-R, GABAB-R1; ↑ 5-HT1A	Sleep enhancement; ↑ NREM & delta waves	[19]
**Chlorogenic acid**	In vitro (Huh-7 HCC cells)	50, 100 µg/mL	↓ Akt signaling; ↓ migration	Anti-cancer	
**Rutin**	In vitro (Huh-7 HCC cells)	50, 100 µg/mL	↑ p53, p16; ↓ ERK	Anti-cancer (apoptosis, senescence)	[20]
**Chlorogenic acid**	In vivo (loperamide-induced constipation)	50–200 mg/kg, 10 days	↑ PGE2; ↑ colonic motility	Laxative	
**Rutin**	In vitro (DPPH)	Identified via LC–MS/MS	Radical scavenging	Antioxidant	[22]
**Rutin**	In vivo (dimethylmercury neurotoxicity)	100 mg/kg, 36 days	↑ Ki67, DCX; ↑ memory	Neuroprotective; memory-enhancing	
**Chlorogenic acid**	In vivo (dimethylmercury neurotoxicity)	100 mg/kg, 36 days	↓ AChE; ↓ oxidative stress	Neuroprotective	[24]
**Chlorogenic acid**	In vitro (HaCaT keratinocytes)	10–300 µg/mL	↓ Tyrosinase; ↓ melanin	Skin-whitening	
**Rutin**	In vitro (porcine elastase assay)	10–400 µg/mL	Elastase inhibition	Anti-wrinkle; skin-firming	[26]

**Chlorogenic acid**	In vitro (DPPH)	HPLC-UV (41.23% MeOH extract)	Radical scavenging	Antioxidant	
**Rutin**	In vivo (scopolamine-induced memory impairment)	125–375 mg/kg, 3 weeks	↓ AChE; ↑ ACh	Neuroprotective; memory enhancement	[27]
**Chlorogenic acid**	In vivo (scopolamine model)	125–375 mg/kg, 3 weeks	↑ Learning & memory performance	Cognitive improvement	
**Rutin**	In vivo (HFD-induced diabetic mice)	Identified via UPLC–QTOF/MS	Neuroprotection; antioxidant modulation	Cognitive enhancement	[29]
**Chlorogenic acid, Rutin**	In vitro (Xanthine oxidase inhibitory assay)	Dose-dependent XO inhibition	Antioxidant; xanthine oxidase inhibition	Antihyperuricemic activity	[34]
**Rutin, Chlorogenic acid**	In vitro (RAW 264.7 macrophages)	Identified via HPLC	Antioxidant; suppression of pro-inflammatory mediators	Anti-inflammatory	

Supplementary Table S5. Representative bioactive compounds isolated from *Dendropanax morbifera* and their reported biological activities and mechanisms of action

Compound	Experimental Model	Experimental Condition/Identification	Key Mechanism	Bioactivity	Reference
**3,5-Dicaffeoylquinic acid**	*In vitro* (RAW 264.7)	LPS-induced inflammation	NF-κB inhibition; ↓ NO & ROS production	Anti-inflammatory	[1]
	In vitro (A549)	250 μg/mL treatment	↑ ROS; p38 MAPK activation; Bcl-2 suppression	Anti-lung cancer	
**Orientin**	*In vivo* (HFD-induced diabetic mice)	Identified via UPLC-QTOF/MS	Neuroprotection: Antioxidant	Cognitive enhancement	[2]
**Isoorientin**	*In vivo* (HFD-induced diabetic mice)	Identified via UPLC-QTOF/MS	Neuroprotection; Antioxidant	Cognitive enhancement	
**Luteolin-7-O-rutinoside**	*In vivo* (HFD-induced diabetic mice)	Identified via UPLC-QTOF/MS	Neuroprotection; Antioxidant	Cognitive enhancement	
**(3S)-Falcarinol**	*In vitro* (Classical Complement Pathway Assay)	IC_{50} = 87.3 μM	Complement inhibition	Anticomplement activity	[3]
**(3S,8S)-Falcarindiol**	*In vitro* (Classical Complement Pathway Assay)	IC_{50} = 15.2 μM	Complement inhibition	Anticomplement activity	
**(3S)-Diynene**	*In vitro* (Classical Complement Pathway Assay)	IC_{50} = 39.8 μM	Complement inhibition	Anticomplement activity	
**Dendropanoxide**	*In vivo* (Streptozotocin-induced diabetic rats)	30, 60, 100 mg/kg for 14 days	↓ Blood glucose, ↑ Insulin, ↓ Lipid levels (cholesterol, triglycerides)	Antidiabetic, hypoglycemic	[4]

	In vivo (Normal rat)	30, 60, 100 mg/kg for 14 days	No metabolic changes observed	No effect in normoglycemic rats	
	In vitro (NRK-52E kidney epithelial cells)	5, 10 μg/mL treatment	↓ Apoptosis (↓ Bax, ↑ Bcl-2, ↓ Cleaved-PARP); ↓ ROS production	Nephroprotective; anti-apoptotic	
	In vivo (Cisplatin-induced acute kidney injury in rats)	5, 10 mg/kg for 7 days	↓ Oxidative stress (↑ SOD, ↓ MDA, ↓ 8-OHdG): ↓ Inflammatory cytokines (TNF-α, IL-6, IL-1β); ↑ AMPK/mTOR signaling	Nephroprotective; anti-inflammatory	
**Quercetin**	*In vitro* (RAW 264.7)	Identified via HPLC	Antioxidant; anti-inflammatory properties	↓ Pro-inflammatory mediators	[6]
	In vitro (Oligodendrocyte precursor cells, OPC)	1:1000 dilution of DM leaf EtOH extract	↑ MBP: ↓ ID2 expression; ↑ ERK1/2 phosphorylation	Oligodendrocyte differentiation myelination enhancement	[16]
	In vivo (Loperamide-induced constipation in rats)	50, 100, 200 mg/kg for 10 days	↓ AQP3 expression; ↑ Fecal water content	Laxative; stool hydration	[24]
	In vitro (HT22 hippocampal neuronal cells)	1–10 μM treatment	↑ Nrf2/HO-1 activation, ↓ ROS, ↓ Apoptosis-inducing factor (AIF) translocation	Neuroprotective; anti-apoptotic	[33]
**(+)-Catechin**	*In vitro* (RAW 264.7)	Identified via HPLC	Antioxidant; anti-inflammatory properties	↓ Pro-inflammatory mediators	
**Ferulic acid**	*In vitro* (RAW 264.7)	Identified via HPLC	Antioxidant; anti-inflammatory properties	↓ Pro-inflammatory mediators	[6]
**Myricetin**	*In vitro* (RAW 264.7)	Identified via HPLC	Antioxidant anti-inflammatory properties	↓ Pro-inflammatory mediators	
**(10E)-9,16-Dihydroxyoctadeca-10,17-dien-12,14-diynoate**	*In vitro* (B16-F10 melanoma cells)	1, 3, 10 μM treatment	PKA/CREB inhibition; p38 MAPK inhibition; MITF suppression	Anti-melanogenic (↓ Tyrosinase, TRP-1, TRP-2, MITF)	[7]

**α-Amyrin**<br>**β-Amyrin**	*In vitro* (RAW 264.7)	LPS-induced inflammation	NF-κB/MAPK inhibition; ↓ iNOS & COX-2 expression	Anti-inflammatory	[12]
**Syringin**	In vivo (STZ-induced diabetic rats)	25 mg/kg/day for 28 days	↓ Renal fibrosis markers (Collagen-1, Fibronectin, α-SMA)<br>↓ ROS	Anti-fibrotic; antioxidant	[15]
	In vitro (DPPH assay)	Identified via LC-MS/MS	Radical scavenging activity	Antioxidant	[25]
	In vivo (Scopolamine-induced memory impairment in mice)	125, 250, 375 mg/kg for 3 weeks	↓ Oxidative stress<br>(↓ MDA, ↑ CAT, SOD)	Antioxidant; neuroprotective	[34]
**Dihydroconiferyl Ferulate**	In vitro (MDA-MB-231, MCF-7 breast cancer cells)	50 μM treatment	↓ Nuclear EGFR interction<br>↓ Stat3 interaction<br>↓ c-Myc expression	Anti-breast cancer stem cell (↓ Mammosphere formation, ↑ Apoptosis)	[18]
**Rosmarinic acid**	In vitro (Huh-7 hepatocellular carcinoma cells)	50, 100 μg/mL treatment	↑ Apoptotic pathway activation	Anti-cancer<br>(↑ Apoptosis)	[22]
**Methyl (10E,9R,16R)-16-acetoxy-9-hydroxyoctadeca-10,17-dien-12,14-diynoate**	In vitro (HepG2 cells)	10 μM treatment	↑ PPAR-α activation; ↑ Sirt1 activation; ↑ AMPK activation	Lipid metabolism regulation	[25]
**Methyl (10E,9R,16S)-9,16-dihydroxyoctadeca-10-en-12,14-diynoate**	In vitro (HepG2 cells)	10 μM treatment	↑ PPAR-α; ↑ AMPK activation	Anti-metabolic syndrome	

**Methyl (10Z,9R,16S)-9,16-dihydroxyoctadeca-10,17-dien-12,14-diynoate**	In vitro (HepG2 cells)	10 μM treatment	↑ PPAR-α; ↑ AMPK activation	Lipid metabolism regulation	
**6-Hydroxyluteolin 7-O-laminaribioside**	In vitro (DPPH assay)	Identified via LC-MS/MS	Radical scavenging activity	Antioxidant	
**Schaftoside**	In vitro (DPPH assay)	Identified via LC-MS/MS	Radical scavenging activity	Antioxidant	
**Kaempferol-3-O-rutinoside**	In vitro (DPPH assay)	Identified via LC-MS/MS	Radical scavenging activity	Antioxidant	
**cis-6-Oxogeran-4-enyl-10-oxy-O-β-arabinopyranosyl-4′-O-β-arabinopyranosyl-2″-octadec-9‴,12‴,15‴-trienoate**	In vitro (DPPH, NO scavenging, FRAP assays)	10, 25, 50, 100 μg/mL treatment	Free radical scavenging, reducing power	Antioxidant	[28]
**Geran-3(10)-enyl-1-oxy-O-β-arabinopyranosyl-4′-O-β-arabinopyranosyl-2″-octadec-9‴,12‴,15‴-trienoate**	In vitro (DPPH, NO scavenging, FRAP assays)	10, 25, 50, 100 μg/mL treatment	Free radical scavenging, reducing power	Antioxidant	
**Geranilan-8-oxy-O-α-d-xylopyranosyl-2′-n-octadec-9″,12″,15″-trienoate**	In vitro (DPPH, NO scavenging, FRAP assays)	10, 25, 50, 100 μg/mL treatment	Mild antioxidant activity	Weak antioxidant	

**1-Cyclohex-2′,5′-dienyl 1-cyclohexylethanol-O-β-d-xylopyranoside**	In vitro (DPPH, NO scavenging, FRAP assays)	10, 25, 50, 100 μg/mL treatment	Mild antioxidant activity	Weak antioxidant	
**Caffeic acid**	In vitro (DPPH assay)	Identified via HPLC-UV, extracted with 41.23% methanol at 88.61°C for 1.86 h	Radical scavenging activity	Antioxidant	[29]
**(9Z,16S)-16-Hydroxy-9,17-octadecadiene-12,14-diynoic acid**	In vitro (HepG2 cells)	50 μg/mL treatment	↓ Triglyceride biosynthesis; ↑ Lipid metabolism regulation	Anti-obesity; lipid metabolism modulation	[30]
**(9Z,16S)-9,17-Octadecadiene-12,14-diyne-1,16-diol**	In vitro (HepG2 cells)	50 μg/mL treatment	↓ Triglyceride accumulation	Anti-obesity	
**(9Z,16S)-16-Hydroxy-19-methyl-9,17-octadecadiene-12,14-diynoic acid**	In vitro (HepG2 cells)	50 μg/mL treatment	↓ Triglyceride synthesis; ↑ AMPK activation	Lipid metabolism regulation	
**(9Z,16S)-16-Hydroxy-9,17-octadecadiene-12,14-diynoic acid (HOD)**	In vitro (HepG2, 3T3-L1 adipocytes)	10–30 μg/mL treatment	↑ AMPK activation; ↓ Triglyceride biosynthesis	Anti-obesity; lipid metabolism regulation	[31]
**(9Z,16S)-16-Hydroxy-9,17-octadecadiene-12,14-diynoic acid (HOD)**	In vivo (HFD-induced obese mice)	250, 500 mg/kg for 8 weeks	↓ Body weight; ↓ Liver steatosis; ↑ AMPK phosphorylation	Anti-obesity; hepatoprotective	
**Isoquercitrin**	In vitro (HT22 hippocampal neuronal cells)	10–100 μM treatment	↑ Nrf2/HO-1 activation, ↓ ROS, ↓ Mitochondrial dysfunction	Neuroprotective; anti-oxidative	[33]

Reference

1. Xu, F.; Valappil, A.K.; Zheng, S.; Zheng, B.; Yang, D.; Wang, Q. 3,5-DCQA as a Major Molecule in MeJA-Treated Dendropanax morbifera Adventitious Root to Promote Anti-Lung Cancer and Anti-Inflammatory Activities. *Biomolecules* **2024**, *14*, DOI 10.3390/biom14060705..
2. Kim, J.M.; Park, S.K.; Guo, T.J.; Kang, J.Y.; Ha, J.S.; Lee, D.S.; Lee, U.; Heo, H.J. Anti-amnesic effect of Dendropanax morbifera via JNK signaling pathway on cognitive dysfunction in high-fat diet-induced diabetic mice. *Behavioural Brain Research* **2016**, *312*, 39, DOI 10.1016/j.bbr.2016.06.013..
3. Chung, I.; Song, H.; Kim, S.; Moon, H. Anticomplement activity of polyacetylenes from leaves of Dendropanax morbifera Leveille. *Phytotherapy Research* **2010**, *25*, 784, DOI 10.1002/ptr.3336..
4. Moon, H. Antidiabetic effects of dendropanoxide from leaves of Dendropanax morbifera Leveille in normal and streptozotocin-induced diabetic rats. *Hum Exp Toxicol* **2010**, *30*, 870, DOI 10.1177/0960327110382131..
5. Lee, D.; Kim, J.; Han, Y.; Park, K.I. Antihyperuricemic Effect of Dendropanax morbifera Leaf Extract in Rodent Models. *Evidence-Based Complementary and Alternative Medicine* **2021**, *2021*, 1, DOI 10.1155/2021/3732317..
6. Hyun, T.K.; Ko, Y.; Kim, E.; Chung, I.; Kim, J. Anti-inflammatory activity and phenolic composition of Dendropanax morbifera leaf extracts. *Industrial Crops and Products* **2015**, *74*, 263, DOI 10.1016/j.indcrop.2015.05.002..
7. Park, J.U.; Yang, S.Y.; Guo, R.H.; Li, H.X.; Kim, Y.H.; Kim, Y.R. Anti-Melanogenic Effect of Dendropanax morbiferus and Its Active Components via Protein Kinase A/Cyclic Adenosine Monophosphate-Responsive Binding Protein- and p38 Mitogen-Activated Protein Kinase-Mediated Microphthalmia−Associated Transcription Factor Downregulation. *Front Pharmacol* **2020**, *11*, DOI 10.3389/fphar.2020.00507..
8. Youn, J.S.; Kim, Y.; Na, H.J.; Jung, H.R.; Song, C.K.; Kang, S.Y.; Kim, J.Y. Antioxidant activity and contents of leaf extracts obtained from Dendropanax morbifera LEV are dependent on the collecting season and extraction conditions. *Food Sci Biotechnol* **2018**, *28*, 201, DOI 10.1007/s10068-018-0352-y..
9. Yang, Y.J.; Song, J.; Yang, J.; Kim, M.J.; Kim, K.Y.; Kim, J.; Jin, Y.B.; Kim, W.H.; Kim, S.; Kim, K.; Park, K.I.; Lee, H. Anti-Periodontitis Effects of Dendropanax morbiferus H.Lév Leaf Extract on Ligature-Induced Periodontitis in Rats. *Molecules* **2023**, *28*, DOI 10.3390/molecules28020849..
10. Park, S.; Karthivashan, G.; Ko, H.M.; Cho, D.; Kim, J.; Cho, D.J.; Ganesan, P.; Su-Kim, I.; Choi, D. Aqueous Extract of Dendropanax morbiferus Leaves Effectively Alleviated Neuroinflammation and Behavioral Impediments in MPTP-Induced Parkinson's Mouse Model. *Oxidative Medicine and Cellular Longevity* **2018**, *2018*, DOI 10.1155/2018/3175214..
11. Hwang, C.E.; Kim, S.C.; Cho, C.S.; Song, W.Y.; Joo, O.S.; Cho, K.M. Comparison of chlorogenic acid and rutin contents and antioxidant activity of Dendropanax morbiferus extracts according to ethanol concentration. *Korean J Food Preserv* **2020**, *27*, 880, DOI 10.11002/kjfp.2020.27.7.880..
12. Kim, K.J.; Youn, J.S.; Kim, Y.; Kim, J.Y. Comparisons of the Anti-Inflammatory Activity of Dendropanax morbifera LEV Leaf Extract Contents Based on the Collection Season and Concentration of Ethanol as an Extraction Solvent. *Applied Sciences* **2020**, *10*, DOI 10.3390/app10238756..
13. Eom, T.; Ko, G.; Kim, K.C.; Kim, J.; Unno, T. Dendropanax morbifera Leaf Extracts Improved Alcohol Liver Injury in Association with Changes in the Gut Microbiota of Rats. *Antioxidants* **2020**, *9*, DOI 10.3390/antiox9100911..
14. Eom, T.; Kim, K.C.; Kim, J. Dendropanax morbifera Leaf Polyphenolic Compounds: Optimal Extraction Using the Response Surface Method and Their Protective Effects against Alcohol-Induced Liver Damage. *Antioxidants* **2020**, *9*, DOI 10.3390/antiox9020120..

15. Sachan, R.; Kundu, A.; Dey, P.; Son, J.Y.; Kim, K.S.; Lee, D.E.; Kim, H.R.; Park, J.H.; Lee, S.H.; Kim, J.; Cao, S.; Lee, B.M.; Kwak, J.H.; Kim, H.S. Dendropanax morbifera Protects against Renal Fibrosis in Streptozotocin-Induced Diabetic Rats. *Antioxidants* **2020**, *9*, DOI 10.3390/antiox9010084..
16. Kim, J.; Yoon, J.; Sugiura, Y.; Lee, S.; Park, J.; Song, G.; Yang, H. Dendropanax morbiferus leaf extract facilitates oligodendrocyte development. *R Soc open sci* **2019**, *6*, DOI 10.1098/rsos.190266..
17. Choi, H.; Park, D.; Song, S.; Yoon, I.; Cho, S. Development and Validation of a HPLC-UV Method for Extraction Optimization and Biological Evaluation of Hot-Water and Ethanolic Extracts of Dendropanax morbifera Leaves. *Molecules* **2018**, *23*, DOI 10.3390/molecules23030650..
18. Ko, Y.; Liu, R.; Sun, H.; Yun, B.; Choi, H.S.; Lee, D. Dihydroconiferyl Ferulate Isolated from Dendropanax morbiferus H.Lév. Suppresses Stemness of Breast Cancer Cells via Nuclear EGFR/c-Myc Signaling. *Pharmaceuticals* **2022**, *15*, DOI 10.3390/ph15060664..
19. Awais, M.; Akter, R.; Boopathi, V.; Ahn, J.C.; Lee, J.H.; Mathiyalagan, R.; Kwak, G.; Rauf, M.; Yang, D.C.; Lee, G.S.; Kim, Y.; Jung, S. Discrimination of Dendropanax morbifera via HPLC fingerprinting and SNP analysis and its impact on obesity by modulating adipogenesis- and thermogenesis-related genes. *Front Nutr* **2023**, *10*, DOI 10.3389/fnut.2023.1168095..
20. Ko, K.; Ahn, Y.; Cheon, G.Y.; Suh, H.J.; Cho, Y.J.; Park, S.; Hong, K. Effects of Dendropanax morbiferus Leaf Extract on Sleep Parameters in Invertebrate and Vertebrate Models. *Antioxidants* **2023**, *12*, DOI 10.3390/antiox12101890..
21. Park, J.U.; Kang, B.Y.; Kim, Y.R. Ethyl Acetate Fraction from Dendropanax morbifera Leaves Increases T Cell Growth by Upregulating NF-AT-Mediated IL-2 Secretion. *Am J Chin Med* **2018**, *46*, 453, DOI 10.1142/s0192415x18500234..
22. Hyun, T.K.; Kim, M.; Lee, H.; Kim, Y.; Kim, E.; Kim, J. Evaluation of anti-oxidant and anti-cancer properties of Dendropanax morbifera Léveille. *Food Chemistry* **2013**, *141*, 1947, DOI 10.1016/j.foodchem.2013.05.021..
23. Piao, D.; Youn, I.; Huynh, T.; Kim, H.W.; Noh, S.G.; Chung, H.Y.; Oh, D.; Seo, E.K. Identification of New Polyacetylenes from Dendropanax morbifera with PPAR-α Activity Study. *Molecules* **2024**, *29*, DOI 10.3390/molecules29245942..
24. Na, J.; Lee, K.H.; Kim, E.; Hwang, K.; Na, C.; Kim, S. Laxative Effects of a Standardized Extract of Dendropanax morbiferus H. Léveille Leaves on Experimental Constipation in Rats. *Medicina* **2021**, *57*, DOI 10.3390/medicina57111147..
25. 김민정; Kim, M.J.; Son, J.D.; Yang, Y.J.; Heo, J.W.; Lee, H.J.; Park, K.I. LC-MS/MS analysis and antioxidant activity of Dendropanax morbiferus extract. , DOI 10.14374/HFS.2024.32.3.235..
26. Kim, W.; Yoo, D.Y.; Jung, H.Y.; Kim, J.W.; Hahn, K.R.; Kwon, H.J.; Yoo, M.; Lee, S.; Nam, S.M.; Yoon, Y.S.; Kim, D.W.; Hwang, I.K. Leaf extracts from Dendropanax morbifera Léveille mitigate mercury-induced reduction of spatial memory, as well as cell proliferation, and neuroblast differentiation in rat dentate gyrus. *BMC Complement Altern Med* **2019**, *19*, DOI 10.1186/s12906-019-2508-6..
27. Hoang, H.T.; Park, J.; Kim, S.; Moon, J.; Lee, Y. Microwave-Assisted Dendropanax morbifera Extract for Cosmetic Applications. *Antioxidants* **2022**, *11*, DOI 10.3390/antiox11050998..
28. Chung, I.; Kim, S.; Kwon, C.; Kim, S.; Yang, Y.; Kim, J.; Ali, M.; Ahmad, A. New Chemical Constituents from the Bark of Dendropanax morbifera Leveille and Their Evaluation of Antioxidant Activities. *Molecules* **2019**, *24*, DOI 10.3390/molecules24213967..
29. Zhang, M.; Bu, T.; Liu, S.; Kim, S.; Durazzo, A. Optimization of Caffeic Acid Extraction from Dendropanax morbifera Leaves Using Response Surface Methodology and Determination of Polyphenols and Antioxidant Properties. **2021**, DOI 10.3390/10.3390/horticulturae7110491..
30. Kim, M.; Kang, M.; Lee, S.; Kim, D.; Jang, H.; An, J.H.; Lee, H.; Ryu, H.W.; Oh, S. Polyacetylene (9Z,16S)-16-hydroxy-9,17-octadecadiene-12,14-diynoic acid in Dendropanax morbifera leaves. *Food Bioscience* **2021**, *40*, DOI 10.1016/j.fbio.2021.100878..

31. Kang, M.; Kwon, E.; Ryu, H.W.; Lee, S.; Lee, J.; Kim, D.; Lee, M.K.; Oh, S.; Lee, H.; Lee, S.U.; Kim, M. Polyacetylene From Dendropanax morbifera Alleviates Diet-Induced Obesity and Hepatic Steatosis by Activating AMPK Signaling Pathway. *Front Pharmacol* **2018**, *9*, DOI 10.3389/fphar.2018.00537..
32. Park, Y.J.; Kim, K.S.; Park, J.H.; Lee, S.H.; Kim, H.R.; Lee, S.H.; Choi, H.B.; Cao, S.; Kumar, V.; Kwak, J.H.; Kim, H.S. Protective effects of dendropanoxide isolated from Dendropanax morbifera against cisplatin-induced acute kidney injury via the AMPK/mTOR signaling pathway. *Food and Chemical Toxicology* **2020**, *145*, DOI 10.1016/j.fct.2020.111605..
33. Park, H.; Kim, H.; Kim, C.Y.; Seo, M.; Baek, S. Synergistic Protection by Isoquercitrin and Quercetin against Glutamate-Induced Oxidative Cell Death in HT22 Cells via Activating Nrf2 and HO-1 Signaling Pathway: Neuroprotective Principles and Mechanisms of Dendropanax morbifera Leaves. *Antioxidants (Basel)* **2021**, *10*, 554. doi: 10.3390/antiox10040554, DOI 10.3390/antiox10040554..
34. Kim, S.B.; Ryu, H.Y.; Nam, W.; Lee, S.M.; Jang, M.R.; Kwak, Y.G.; Kang, G.I.; Song, K.S.; Lee, J.W. The Neuroprotective Effects of Dendropanax morbifera Water Extract on Scopolamine-Induced Memory Impairment in Mice. *IJMS* **2023**, *24*, DOI 10.3390/ijms242216444..
35. Yun, J.; Kim, S.; Kim, Y.; Choi, E.J.; You, J.; Cho, E.; Yoon, J.; Kwon, E.; Kim, H.; Jang, J.; Park, J.; Che, J.; Kang, B. Preclinical study of safety of Dendropanax morbifera Leveille leaf extract: General and genetic toxicology. *Journal of Ethnopharmacology* **2019**, *238*, DOI 10.1016/j.jep.2019.111874..
